# Rosmarinic and Glycyrrhetinic Acid-Modified Layered Double Hydroxides as Functional Additives for Poly(Lactic Acid)/Poly(Butylene Succinate) Blends

**DOI:** 10.3390/molecules28010347

**Published:** 2023-01-01

**Authors:** Francesca Cicogna, Elisa Passaglia, Matilde Benedettini, Werner Oberhauser, Randa Ishak, Francesca Signori, Serena Coiai

**Affiliations:** 1 National Research Council-Institute for the Chemistry of OrganoMetallic Compounds (CNR-ICCOM), SS Pisa, Via Moruzzi 1, 56124 Pisa, PI, Italy; 2 National Research Council-Institute for the Chemistry of OrganoMetallic Compounds (CNR-ICCOM), Via Madonna del Piano 10, 50019 Sesto Fiorentino, FI, Italy; 3 Department of Civil and Industrial Engineering, University of Pisa, Largo L. Lazzarino 1, 56122 Pisa, PI, Italy

**Keywords:** rosmarinic acid, glycyrrhetinic acid, layered double hydroxides, PLA/PBS blend, antioxidant, antibacterial, controlled migration

## Abstract

Immobilizing natural antioxidant and biologically active molecules in layered double hydroxides (LDHs) is an excellent method to retain and release these substances in a controlled manner, as well as protect them from thermal and photochemical degradation. Herein, we describe the preparation of host–guest systems based on LDHs and rosmarinic and glycyrrhetinic acids, two molecules obtained from the extraction of herbs and licorice root, respectively, with antioxidant, antimicrobial, and anti-inflammatory properties. Intercalation between the lamellae of the mono-deprotonated anions of rosmarinic and glycyrrhetinic acid (RA and GA), alone or in the presence of an alkyl surfactant, allows for readily dispersible systems in biobased polymer matrices such as poly(lactic acid) (PLA), poly(butylene succinate) (PBS), and a 60/40 wt./wt. PLA/PBS blend. The composites based on the PLA/PBS blend showed better interphase compatibility than the neat blend, correlated with increased adhesion at the interface and a decreased dispersed phase size. In addition, we proved that the active species migrate slowly from thin films of the composite materials in a hydroalcoholic solvent, confirming the optimization of the release process. Finally, both host–guest systems and polymeric composites showed antioxidant capacity and, in the case of the PLA composite containing LDH-RA, excellent inhibitory capacity against *E. coli* and *S. aureus*.

## 1. Introduction

Food packaging has the fundamental role of improving the shelf-life of food, facilitating its handling, and protecting it from physicochemical damage during storage or transportation. The protection of foodstuffs from deterioration leads to better quality (to protect health), less waste (with an increase in the shelf-life of the products), and easier distribution management. Many packaging systems made of plastics and developed to preserve foods are designed for single use. Consequently, the impact of polymeric packaging on the environment is currently one of the most concerning problems. To partially overcome this problem, researchers have developed new polymeric materials suitable for packaging that are biodegradable and compostable and that, in some cases, can be obtained from renewable resources. Among the most studied biobased polymers, poly(lactic acid) (PLA) is the one that shows the best-performing characteristics as a substitute for non-biodegradable polymers in the packaging sector. Indeed, PLA is the first biobased polymer commercialized on a large scale that can be transformed into injection-molded objects, films, and coatings [1] and is a food contact material approved by the US Food and Drug Administration. Despite these interesting properties, its use for food packaging is limited because it shows poor ductility and scarce thermal and oxygen barrier properties. Therefore, to improve the PLA performance, PLA plasticization [2], the addition of nanofillers [3] or natural fiber reinforcements [4], and blending with other biopolymers [5,6] have been proposed as efficient methods. For example, a PLA/polyhydroxybutyrate (PHB) blend plasticized with 15 wt.% of acetyl(tributyl citrate) showed higher thermal stability, a higher oxygen barrier, and higher stretchability than PLA [2,7]. Blends between PLA and poly(butylene succinate-co-adipate) (PBSA) (20 wt.%) also showed a good distribution of phases and improved mechanical properties compared to PLA [5]. Poly(butylene succinate) (PBS) is another compostable and biobased polyester that is now obtainable from renewable sources. It is characterized by high ductility, high thermal resistance and deflection temperature, and biocompatibility [8], which make it attractive when used in blends with PLA [9,10]. For example, a PLA/PBS (80/20 wt./wt.) blend plasticized with an isosorbide diester (15 wt.%) showed higher deformability [6]. Recently, PLA/PBSA (70/30 wt./wt.) blends containing 3–6 wt.% of thymol were also investigated as a material for active packaging to preserve fresh bread [11]. In addition, Qin et al. [12] developed PLA/polycaprolactone (PCL)/cinnamaldehyde films with antimicrobial activity and used these films to package fresh bread.

The latter examples introduce the concept of active packaging (AP). AP systems are designed to “deliberately incorporate components that could release or absorb substances into the packaged food or the surrounding environment,” as defined in European Regulation No. 450/2009 [13]. AP materials are thus “intended to extend the shelf life or maintain or improve the condition of packaged food.” These improvement effects can be achieved, for example, by adding active molecules, such as antimicrobial agents or antioxidants, which are slowly released from the packaging without compromising the quality, safety, and integrity of food products [14,15]. The primary physical phenomenon exploited in AP systems is mass transport (adsorption and migration) through polymer films of active substances, but this aspect may pose some concerns. For example, due to poor compatibility with polymer chains, antioxidants and low-molecular-weight antimicrobial agents dispersed in polymer matrices can easily migrate from the bulk to the surface. In such cases, active molecules are rapidly released without control, limiting their use in the target application.

A widely studied method for achieving better control of the release of active agents is fixing or encapsulating them into nanofillers able to hold them [16]. In these hybrid systems, the migration of active substances can be tuned and optimized based on the kind and extent of their interaction with the nanofiller. Furthermore, low release is generally reached and maintained even after the dispersion of the encapsulated system into a polymeric matrix, transferring the nanofiller properties to the polymer matrix and conferring new properties to the packaging [16,17]. The resulting polymer nanocomposites can thus be suitable for food packaging that maintains food quality over an appropriate timescale. Furthermore, polymer nanocomposites show higher mechanical, thermal, and barrier performance than neat polymers. Therefore, polymer nanocomposites, in the possible case of their use for food packaging, not only have the primary function of containment but also improve the mechanical strength and barrier to gases of packaging, preventing the breakage of the container and the fast deterioration of food products.

Among the different inorganic fillers used to prepare host–guest systems with active agents, layered double hydroxides (LDHs) are one of the best-performing materials. LDHs are anionic clays of positively charged and pillared metal hydroxide lamellae balanced by hydrated anions intercalated between the lamellae [18]. LDHs are materials useful for the uptake, storage, and controlled release of industrially relevant and biologically active anions. For this reason, LDHs have a great variety of applications in cosmetics, packaging, and other sectors as adsorbents, photoluminescence sensors, antimicrobial materials, drug delivery, etc., and in recent years, they have been studied to achieve functional materials for AP [19,20,21,22,23,24]. Interestingly, host–guest structures derived from the intercalation of active substances in LDHs lead to new systems where LDHs control the release kinetics of active molecules and protect them from thermal and light degradation. In the last several years, the evolution of materials suitable for food packaging has marked a clear transition from reinforced and gas barrier oil-based polymer materials to biobased active and functional polymer nanocomposites. This transition can be summarized with the new term “bionanocomposite”, which combines biopolymer and active nano-additives [25]. The addition of different nanofillers to biopolymers can effectively improve the properties of biopolymers and change the rate of degradation [26].

**Figure 1:**
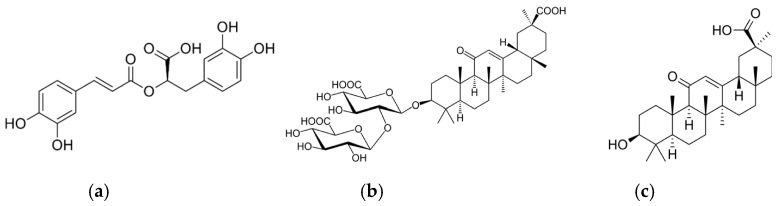
Chemical structure of rosmarinic acid (**a**), glycyrrhizic acid (**b**), and 18β-glycyrrhetinic acid (**c**).

Furthermore, in the last few decades, there has been a growing interest in the use of natural compounds in place of synthetic additives in the food industry [27]. In this direction is “food biopreservation”, which ensures food safety by adding natural antimicrobial agents directly to food. Therefore, the extension of this concept to AP leads to the use of natural antimicrobial or antioxidant ingredients in polymeric materials for food packaging [28]. Among natural antimicrobial and antioxidant agents, those extracted from plants are particularly interesting, and organic acids and their salts have great activity and are thus appealing for the proposed application [16,29,30,31]. Other interesting plant extracts are polyphenols, which have antimicrobial activity and can extend the shelf-life of food products [32,33,34]. Among polyphenolic compounds, rosmarinic acid, commonly found in species of the subfamily Nepetoideae of the Lamiaceae and Boraginaceae, is particularly interesting for its functional properties and biological activities (Figure 1) [35,36,37].

Another organic acid with interesting properties is 18β-glycyrrhetinic acid, a pentacyclic organic triterpenoid aglycone of glycyrrhizin obtained from the hydrolysis of glycyrrhizic acid, which is extracted from licorice (Figure 1) [38,39,40,41]. 18β-Glycyrrhetinic acid has anticancer, anti-inflammatory, antioxidant, antiviral, antiulcer, antidiabetic, hepatoprotective, cardioprotective, and neuroprotective effects [38] and antimicrobial activity against *S. aureus* [42] and Gram-positive bacteria [43]. This acid is mainly used for biomedical and cosmetic applications [44,45,46,47,48,49], and its use for packaging has not been reported yet. However, nanostructured systems based on chitin nanofibrils-nanolignin and entrapped 18β-glycyrrhetinic acid have recently been prepared [50]. These complexes, showing cytocompatibility and anti-inflammatory activity, have been embedded in a PLA matrix or deposited on the surface of extruded PLA-based sheets [51].

Herein, we report the synthesis of host–guest hybrid systems consisting of LDH modified with the mono-deprotonated anions of rosmarinic acid (RA) or 18β-glycyrrhetinic acid (GA). LDH was modified by the anion-exchange method with RA and GA to produce LDH-GA and LDH-RA hybrids. To favor the intercalation of the bulky carboxylic acid anions, the co-intercalation of an alkyl surfactant, dodecyl sulfate (DS), was also investigated, providing the LDH-DS/RA and LDH-DS/GA systems. All hybrids were dispersed in PLA, PBS, and a 60/40 wt./wt. PLA/PBS blend to achieve biocomposite materials for potential use in AP. The structural, thermal, and morphological properties of the composites are discussed. In addition, the antimicrobial activity of PLA composite films containing LDH-RA and LDH-GA against two pathogens commonly causing food spoilage is described. Finally, the controlled migration of RA and GA anions confined in LDH and dispersed in PLA is investigated through contact tests of the composite films in 95% ethanol as a fatty food simulant.

## 2. Results and Discussion

### 2.1. Mono-Deprotonated Rosmarinic and 18β-Glycyrrhetinic Acid-Modified LDHs

Four host–guest systems were prepared via the anion exchange of LDH-NO_3_ with mono-deprotonated anions of rosmarinic acid (RA) or 18β-glycyrrhetinic acid (GA) (LDH-GA and LDH-RA, respectively) and DS, which was co-intercalated in an equimolar amount to the anions of RA and GA, thus obtaining LDH/DS-RA and LDH/DS-GA. This latter method was adopted to favor the intercalation of RA and GA, as the co-intercalation of the alkyl DS chain increases the interlamellar distance and promotes the entry of the bulkier species [52]. In addition, the intercalation of alkyl chains could be advantageous in dispersing the layered filler in polymeric matrices [53].

**Figure 2:**
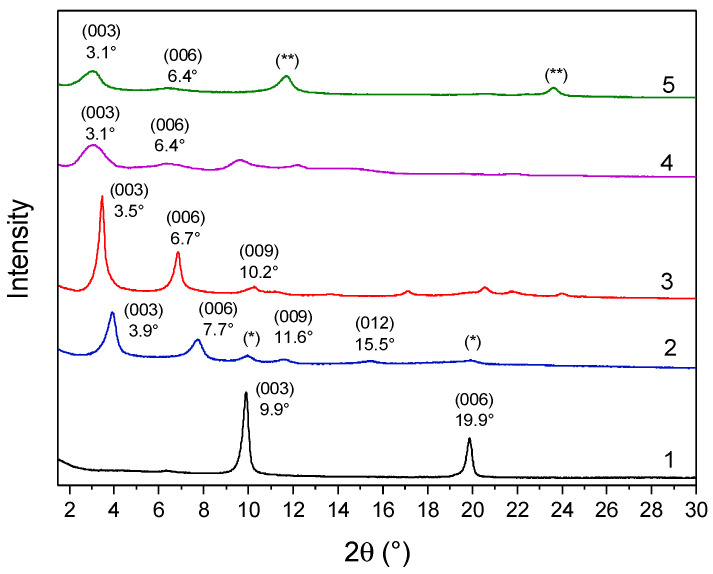
XRD patterns of samples LDH-NO_3_ (1), LDH-RA (2), LDH-DS/RA (3), LDH-GA (4), and LDH-DS/GA (5). Reflections (*) are likely due to a small fraction of unexchanged LDH-NO_3_. Reflection (**) at 11.7° and 23.6° are probably due to the intercalation of carbonate anions that occurred during the preparation or purification of the host–guest system.

The diffraction pattern of LDH-RA compared with that of LDH-NO_3_ shows, as previously reported, that the intercalation of the organic anion was achieved with an increase in the basal spacing from 0.89 nm to 2.26 nm and the maintenance of a high structural order (Figure 2 and Table 1) [24]. Furthermore, the interlamellar space of 1.78 nm (considering 0.48 nm as the thickness of the lamellae [54]) is consistent with the nonplanar geometry of the anion, as previously demonstrated by molecular modeling [55].

The XRD pattern of the co-intercalated LDH-DS/RA (Figure 2) showed intense and narrow signals at 3.5° (003), 6.7° (006), and 11.2° (009), with a basal spacing of 2.52 nm, consistent with the intercalation of alkyl sulfate anions (Table 1) [52,53]. The different basal spacings of LDH-RA and LDH-DS/RA are thus apparently due to the presence of alkyl sulfate anions rearranging in the interlamellar space, likely assuming a monolayer paraffinic-like arrangement [56]. The XRD profile of LDH-GA (Figure 2) shows two diffraction signals at 3.1° and 6.4°, which can be associated with the (003) and (006) reflections with an increase in basal spacing from 0.89 nm to 2.85 nm (Table 1). This finding is consistent with the intercalation of sterically bulky GA anions. Notably, the XRD pattern of LDH-DS/GA (Figure 2) also showed reflections at 3.1° (003) and 6.4° (006) with a basal spacing of 2.85 nm, as in the case of LDH-GA. In this case, the mono-deprotonated anion of GA, which is bulkier than the DS anion, is predominant in determining the gallery’s height.

**Figure 3:**
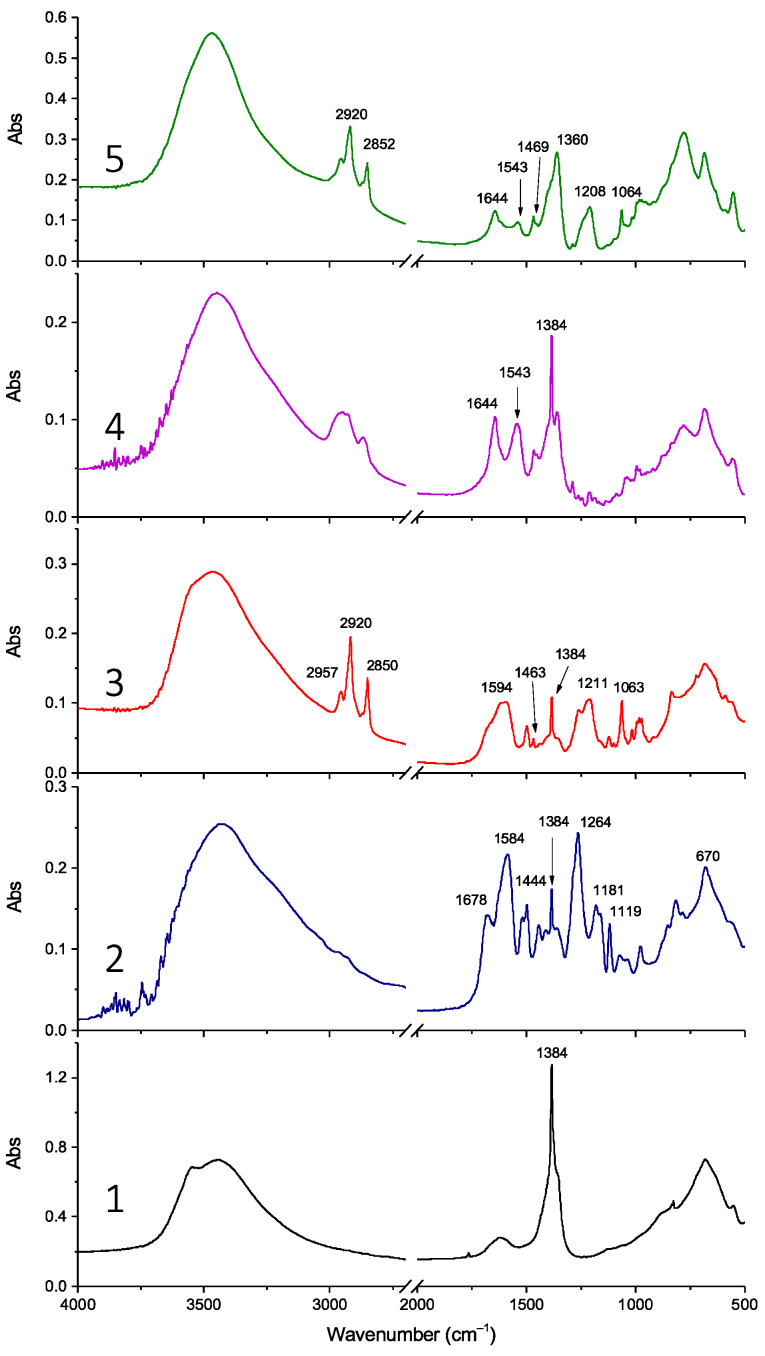
FT-IR spectra of samples LDH-NO_3_ (1), LDH-RA (2), LDH-DS/RA (3), LDH-GA (4), and LDH-DS/GA (5).

FT-IR analysis confirmed the success of anion exchange, showing the incorporation of all organic anions. For example, the FT-IR spectrum of LDH-RA compared with that of LDH-NO_3_ (Figure 3, spectra 1 and 2) shows characteristic bands of the carboxylate anion of rosmarinic acid (i.e., 1678 cm^−1^ (νC=C), 1584 cm^−1^ (ν_as_COO), and 1444 cm^−1^ (νCC, δOH bending in plane) [55]), as previously described [24] and confirmed by the FT-IR spectrum of the sodium salt of rosmarinic acid (Figure S1). In the LDH-DS/RA spectrum (Figure 3, spectrum 3), the C-H stretching modes of DS are recognizable in the high-frequency region at 2957, 2920, and 2850 cm^−1^ [57]. In addition, the spectrum shows asymmetric and symmetric S-O stretching modes of sulfate in the low-frequency region at 1211 and 1063 cm^−1^, respectively, like the spectrum of a DS-only-modified LDH (LDH-DS) (Figure S1).

The spectrum of LDH-DS/RA shows the asymmetric -COO stretching of the carboxylate anion of RA at 1594 cm^−1^ and a narrow band at 1384 cm^−1^ indicative of the presence of the anion. The FT-IR spectrum of LDH-GA (Figure 3, spectrum 4) shows absorbances at 1644 and 1543 cm^−1^ due to the C=O stretching of the ketone group and the asymmetric stretching of the carboxylate anion, respectively, as found for the sodium salt of GA (NaGA) [49] (Figure S1). However, the narrow absorbance at 1384 cm^−1^ in the LDH-GA spectrum reveals that nitrate anions are still present due to incomplete anion exchange between nitrate and carboxylate anions, in agreement with XRD. Finally, the FT-IR spectrum of LDH-DS/GA (Figure 3, spectrum 5) confirms the presence of both DS and GA anions. Specifically, the C-H stretching vibration bands at 2920 and 2852 cm^−1^, the C-H bending mode at 1469 cm^−1^, and the S-O asymmetric and symmetric sulfate stretching modes at 1208 and 1063 cm^−1^ suggest the presence of the DS anion, while the peaks at 1644 and 1543 cm^−1^ confirm that of GA.

TGA thermograms of all LDHs show three main degradation phases (Figure S2), similar to what has been observed previously [24,52,53]. The first phase, from 30 to 200 °C, can be attributed to the loss of water adsorbed on the surface or intercalated between the lamellae; the second weight loss, between 200 and 250 °C, corresponds to the desorption and initial decomposition of organic anions, in agreement with the TGA curves of rosmarinic acid, NaGA, and LDH-DS (Figure S3). The third and final stage, between 250 and 600 °C, is attributable to the complete degradation of organic anions combined with the dehydroxylation of the layers, as observed for LDH-NO_3_ (Figure S3). Finally, at the end of the process, the residue is a mixed oxide of Mg and Al, the amount of which is proportional to the percentage of hydroxide lamellae in the hybrid system (Table 1).

UV-vis spectra of solutions obtained by dissolving small amounts of the modified LDHs in concentrated HCl and diluting with EtOH have been recorded [58]. This treatment dissolves the inorganic layers and keeps the organic anions in the solution, allowing them to be quantified. By constructing calibration curves for both rosmarinic and glycyrrhetinic acids (Figure S4), molar extinction coefficients were calculated for the absorption band at 330 nm for rosmarinic acid and 250 nm for glycyrrhetinic acid, evidencing perfect agreement with values reported in the literature [59,60]. The data obtained (Table 1) confirmed, as shown qualitatively by FT-IR analysis, that the RA content was higher in LDH-RA (37 wt.%) than in the co-intercalated product LDH-(DS/RA) (13 wt.%). A similar result was also obtained for the GA intercalation (45 wt.% for LDH-GA and 13 wt.% for LDH-DS/GA).

### 2.2. PLA, PBS, and PLA/PBS Nanocomposites with Modified LDHs

#### 2.2.1. Structure and Morphology

The four host–guest systems were used to prepare composites with PLA, PBS, and a 60/40 wt./wt. PLA/PBS blend as matrices. The composition of the blend was selected as it is optimized for application in the packaging industry [6,61]: indeed, this polymer ratio increases the elongation at break of PLA by about 40% and significantly improves its impact strength. Composites containing 5 wt.% of modified LDHs were targeted to maximize the content of functional filler and achieve desirable antioxidant and antimicrobial properties. Samples are coded as polymer matrix/modified LDHs (for example, PLA containing LDH-DS/RA is coded PLA/LDH-DS/RA, and the PLA/PBS blend containing LDH-GA is coded PLA/PBS/LDH-GA). The samples were prepared by a two-step solution mixing process: (a) the probe sonication of the modified LDH suspension to promote dispersion and delamination and (b) the dropwise addition of the LDH suspension into the polymer solution. Finally, the resultant materials were compression-molded to yield films.

The molecular weights of PLA and PBS composites with LDH-RA and LDH-DS/RA were higher than those of the neat polymers. Potentially, the organic anions adsorbed on the surface of LDH lamellae protected the polymers from direct contact with Al and Mg ions and Mg-OH basic sites that normally cause PLA degradation [62]. Additionally, RA could act as an antioxidant by limiting the decrease in the molecular weights of PLA and PBS, as observed previously for analogous composites prepared with LDH intercalated with synthetic antioxidants [23]. On the contrary, GA-modified LDHs (i.e., LDH-GA and LDH-DS/GA) yielded a decrease in both Mn and Mw, although of limited magnitude, except for the PLA/LDH-GA sample (Table S1).

**Figure 4:**
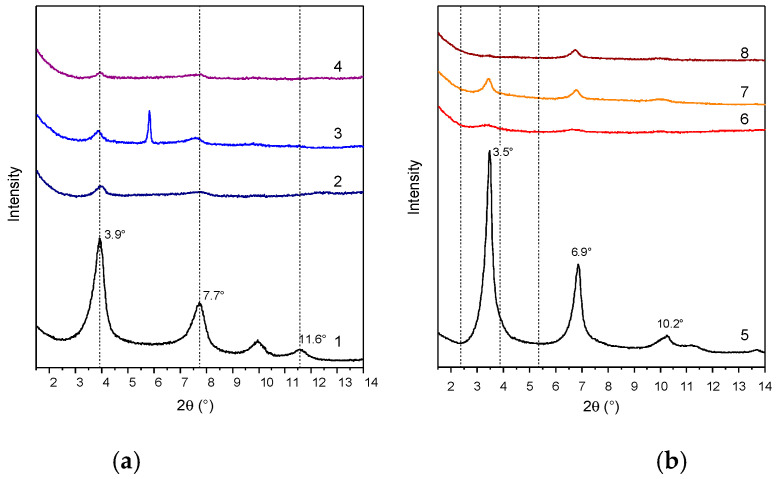
XRD patterns of LDH-RA (1), PLA/LDH-RA (2), PBS/LDH-RA (3), and PLA/PBS/LDH-RA (4) (**a**) and LDH-DS/RA (5), PLA/LDH-DS/RA (6), PBS/LDH-DS/RA (7), and PLA/PBS/LDH-DS/RA (8) (**b**). The signal at 5.8° (2θ) in the XRD pattern of PBS/LDH-RA (3) is due to the presence of impurity.

The degree of dispersion of LDHs in the polymer matrices was evaluated by XRD analysis. XRD patterns of composites in the low-angle region, where the reflections are attributable to the presence of LDH, showed narrow signals at about 3.9° and 7.7° (2θ), corresponding to diffractions (003) and (006) of LDH-RA, thus suggesting the retention, at least in part, of the ordered structure of the filler (Figure 4a). However, this result does not exclude the possibility that a portion of LDH-RA was delaminated. In fact, in the case of the PLA/PBS/LDH-RA composite, the (003) and (006) reflections due to LDH-RA have a very low intensity, suggesting that only a tiny amount of LDH-RA dispersed in the polymer matrix retained its original intercalated shape.

**Figure 5:**
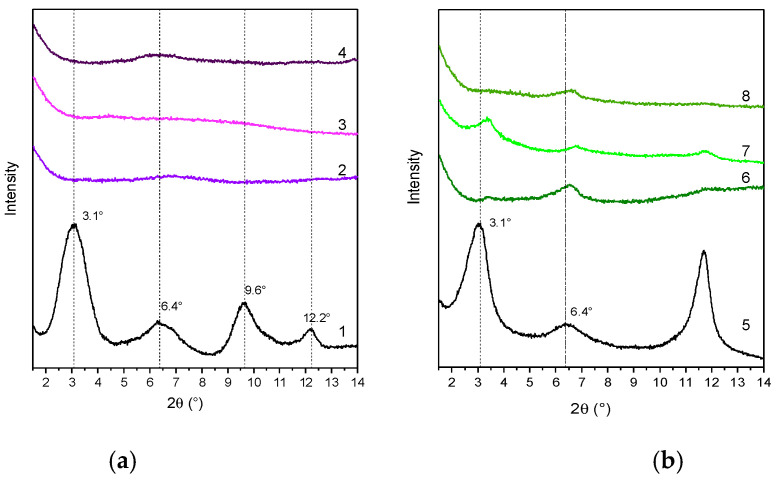
XRD patterns of LDH-GA (1), PLA/LDH-GA (2), PBS/LDH-GA (3), and PLA/PBS/LDH-GA (4) (**a**) and LDH-DS/GA (5), PLA/LDH-DS/GA (6), PBS/LDH-DS/GA (7), and PLA/PBS/LDH-DS/GA (8) (**b**).

The XRD patterns of the PLA/LDH-DS/RA, PBS/LDH-DS/RA, and PLA/PBS/LDH-DS/RA composites (Figure 4b) show weak diffraction signals in the low-angle diffraction zone due to the (003) and (006) LDH-DS/RA reflections. However, in this case, for both PLA/LDH-DS/RA and PBS/LDH-DS/RA composites, the reflections of LDH-DS/RA are shifted to smaller angles than those of the corresponding pure hybrid, suggesting an increase in the interlayer spacing. Moreover, in the case of PLA/LDH-DS/RA, the intensity of these signals is very low, indicating that the fraction of intercalated and ordered lamellae dispersed in PLA is relatively low. For the PLA/PBS/LDH-DS/RA composite, the filler’s basal reflection (003) is not visible, indicating a disordered or exfoliated morphology. These results demonstrate that the co-intercalation of DS anions improved the dispersion ability in the three polymer matrices. Finally, in the case of the composites prepared with LDH-GA and LDH-DS/GA (Figure 5a,b, respectively), the diffraction signals of the fillers are not visible in any of the composites, suggesting a good dispersion of both fillers in all matrices. However, the effect could also be due to the low intensity of the basal diffraction signals of LDH-GA and LDH-DS/GA and the subsequent dilution in the polymer.

Therefore, to clarify these uncertainties, the morphology was studied in more detail by acquiring SEM images of the cryo-fractured surfaces of the composite films. The micrographs of PLA/LDH-RA, acquired at different magnifications, showed a homogeneous fracture morphology in which micrometer-sized and submicrometer-sized particles of LDH-RA (from a few microns to <1 μm) can be observed, appearing as brighter areas (Figure S5). In the case of PLA/LDH-GA, the morphology was homogeneous throughout the fracture, although at higher magnification, some micrometer-sized agglomerates could be observed (Figure S5). This result suggests a better dispersion of LDH-GA than LDH-RA in PLA and confirms the XRD data.

**Figure 6:**
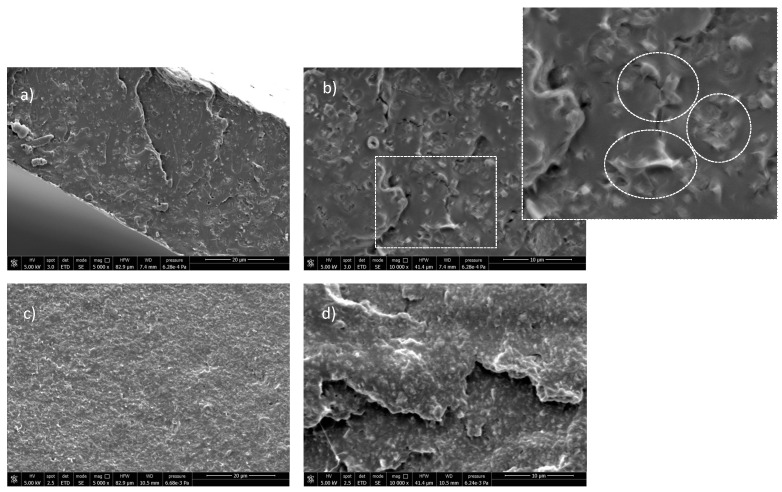
SEM images at different magnifications of PLA/LDH-DS/RA ((**a**,**b**)) and PLA/LDH-DS/GA ((**c**) and (**d**)).

The effect of DS co-intercalation in promoting the dispersion of LDH-DS/RA and LDH-DS/GA in PLA was also investigated. For this purpose, SEM images of the two samples were recorded and compared (Figure 6).

Interestingly, the low-magnification SEM images of PLA/LDH-DS/RA (Figure 6a) showed a relatively homogeneous morphology. At the same time, at higher magnification (Figure 6b), numerous well-dispersed and evenly distributed structures were visible in the polymer phase due to LDH layers. The lamellae may have been well embedded in the polymer and differently oriented from the surface. In the case of the PLA/LDH-DS/GA sample, SEM micrographs did not reveal the presence of LDH-DS/GA (Figure 6c,d), suggesting a good dispersion and distribution of lamellae, down to the nanoscale, and confirming the XRD data.

**Figure 7:**
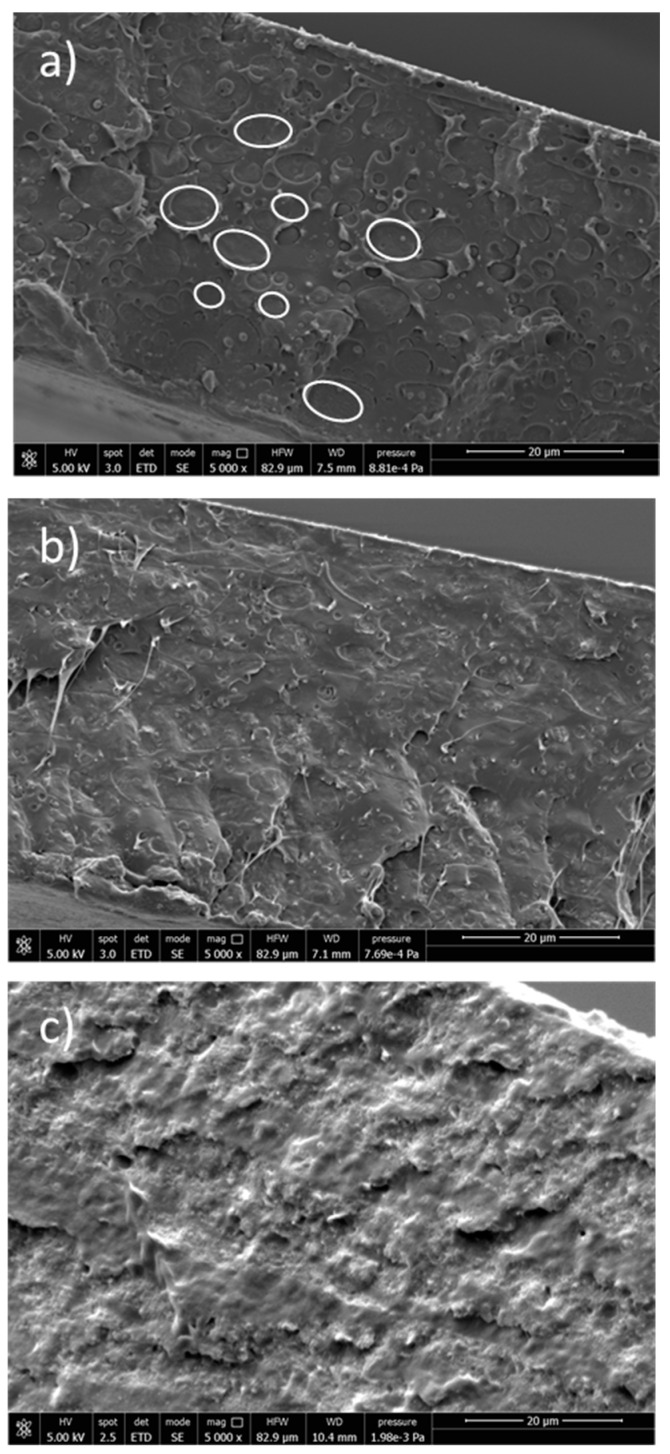
SEM images of PLA/PBS 60/40 wt.% blend (**a**), PLA/PBS/LDH-DS/RA (**b**), PLA/PBS/LDH-DS/GA (**c**).

SEM images of the PLA/PBS mixture (Figure 7a) showed phase separation due to the immiscibility of PLA and PBS [9]. Spherical and nearly spherical PBS droplets with smooth surfaces and variable sizes appeared coarsely dispersed in the PLA matrix, showing poor adhesion between the phases.

The addition of LDH-DS/RA promoted compatibilization between PLA and PBS, with improved adhesion at the interface and reduced PBS droplet size (Figure 7b). This effect was even better in the case of PLA/PBS/LDH-DS/GA, where the phase separation between PLA and PBS completely disappeared (Figure 7c). In addition, no LDH particles were evident in these two composites, confirming their good dispersion. The addition of nanoparticles is reported as a method to manipulate the polymer blend microstructure. Platelet-like nanoparticles, such as anionic and cationic clays, have been found to improve the adhesion between different polymer phases by arranging at the polymer/polymer interface [63,64]. Accordingly, the improved compatibility of the PLA/PBS blend with the addition of LDH-DS/RA and LDH-DS/GA indicates that the lamellae have been arranged at the interface and are exfoliated.

#### 2.2.2. Thermal Properties

**Figure 8:**
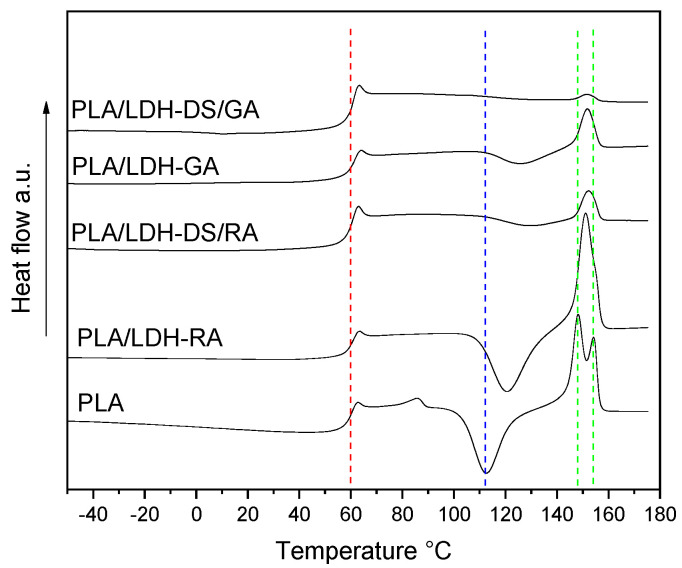
DSC curves (2^nd^ heating) of PLA, PLA/LDH-RA, PLA/LDH-GA, PLA/LDH-DS/RA, and PLA/LDH-DS/GA. The red, blue, and green dashed lines indicate the fundamental transitions of pure PLA: glass transition, cold crystallization, and melting.

The effects of the dispersion of modified LDHs on the thermal properties of the composites were assessed by comparing the thermal transitions of the single-matrix composites with those of the PLA/PBS blend. The T_g_ of all PLA composites was close to that of the neat polymer, even though the cold crystallization process is partially hindered in the composites containing LDH-GA, LDH-DS/RA, and LDH-DS/GA (Figure 8). This behavior is reflected in a lower value of the melting enthalpy in composites than in PLA (Table S2). Furthermore, only one melting peak was observed at an intermediate temperature between the two melting temperatures of PLA. Presumably, the strong interactions between PLA chains and modified lamellae and the presence of dispersed modified LDH prevented the free crystallization of PLA [62].

**Figure 9:**
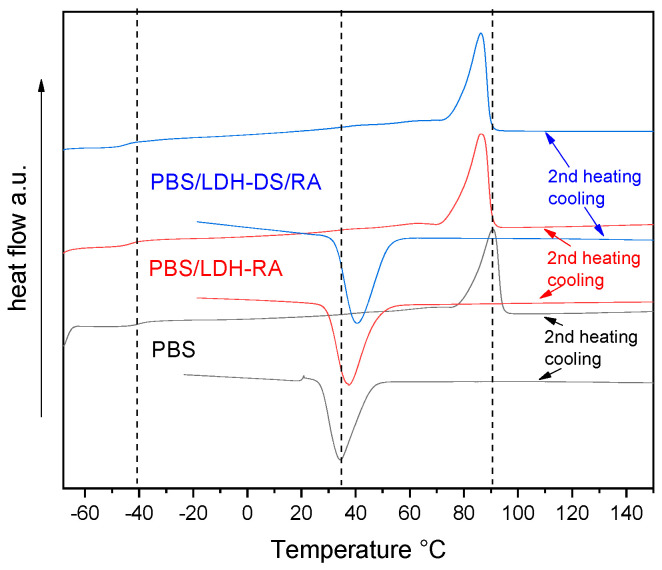
DSC curves (2^nd^ heating and cooling) of PBS, PBS/LDH-RA, and PBS/LDH-DS/RA. Dashed lines indicate the fundamental transitions of pure PBS: glass transition, crystallization, and melting.

In the PBS composites with LDH-RA and LDH-DS/RA, the T_g_ value is like that of PBS (Figure 9 and Table S2). On the contrary, T_c_ shifted to a higher temperature of about 5–10 °C due to the nucleation effect of the nanofillers being more consistent for DS co-intercalated LDH. As a result, the crystallinity of these samples increased compared with PBS but melting occurred at a lower temperature than in PBS due to the formation of less ordered and packed crystallites (Figure 9 and Table S2). Similar results were obtained for composites with LDH/GA and LDH-DS/GA.

**Figure 10:**
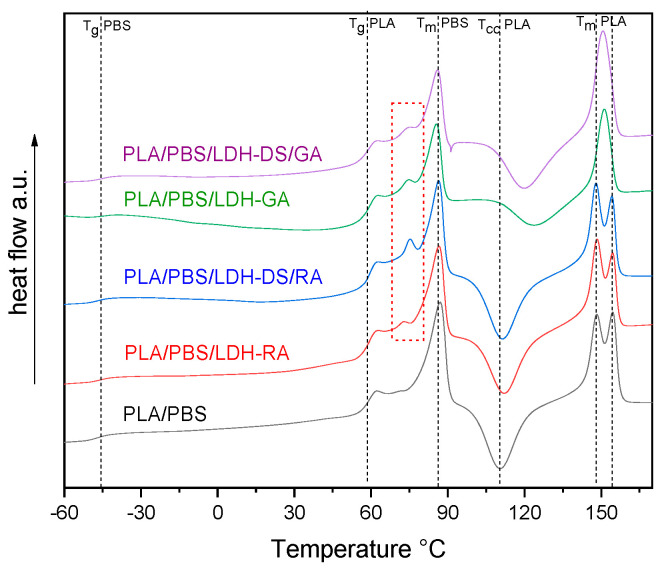
DSC curves (2^nd^ heating) of PLA/PBS blend and its composites. The dashed black lines indicate the fundamental transitions of the PLA/PBS blend, while the dashed red square indicates a new endothermic transition not present in the PLA/PBS blend.

DSC analysis of PLA/PBS blend evidenced the typical behavior of scarcely compatible polymers, showing all the transitions of PLA and PBS alone (Figure S6). The DSC curves of the PLA/PBS composites showed unchanged T_g_ values for both polymer phases (Figure 10), confirming the immiscibility of polymers. However, variations in the cold crystallization temperature and the shape of the PLA melting peak are evident, especially in the composites containing LDH-GA and LDH-DS/GA. Indeed, the T_cc_ of the PLA phase was about 14 and 10 °C higher than that of the PLA/PBS blend, respectively, and the peak was quite broad compared with that of the pure matrix. This shift and the peak broadening suggest that the cold crystallization of PLA was partially hindered by the increased number of interfaces that strengthened the adhesion between the two polymer phases. This evidence confirms the morphological analysis results that show a finer dispersion of the PBS phase for all composites containing the co-intercalated LDH. Finally, in the samples containing LDH-GA and LDH-DS/GA, there was only one melting peak of the PLA phase at a temperature between the two peaks of the PLA/PBS blend. Furthermore, the melting temperature of the PBS phase in all composites does not change from that of the PLA/PBS blend, although a decrease in the melting enthalpy is observed in the composites containing LDH-GA and LDH-DS/GA. Interestingly, an additional endothermic transition is observed for all composites at about 72–75 °C (red dashed square in Figure 10). This new transition, which may be associated with a third phase, is located between the glass transition of PLA and the melting of PBS. LDH embedded in the PLA/PBS matrix probably forms intercalated macroaggregate structures (observed in the SEM micrographs) in which some polymer fractions crystallize, and LDH acts as a heterogeneous nucleating agent.

The curves for PLA/PBS composites obtained during cooling (Figure S7) showed the peak of PBS crystallization. In the case of the PLA/PBS blend, the crystallization peak is broad and centered at 41 °C (7 °C higher than that of PBS alone: see dashed line in Figure S7) with a shoulder at about 50 °C. This behavior confirms PLA/PBS’s mutual nucleation/co-crystallization effects. In the composites, the nucleation effect was even more evident, and in that containing LDH-RA, the PBS crystallization peak was narrower than in the blend. For these samples, the third crystalline phase is probably located at the interphase and, given the shape of the PBS crystallization peak, is probably PLA.

From these results, all modified LDHs can interact with the two polymer phases of the matrix and change their morphology by reducing the size of the PBS. In composites containing LDH-RA and LDH-DS/RA, the modified LDHs preferentially interact with PLA. In composites containing LDH-GA and LDH-DS/GA, interaction occurs with both phases, suggesting an arrangement of the modified LDHs at the interface between them. Overall, the LDH appears to act as a compatibilizer.

#### 2.2.3. Migration Test

The study of the migration behavior of active molecules from polymer films is significant because it dramatically influences one of the most important aspects of AP, namely, the maintenance of functional properties over time. Therefore, among all of the composites prepared in this work, the migration of RA and GA from PLA/LDH-RA and PLA/LDH-GA films was studied by suspending them in an EtOH/H_2_O (95/5 *v*/*v*) solution and by recording the UV-vis absorption spectra over time. The EtOH/H_2_O solution was chosen because it is a fatty food simulant [65].

**Figure 11:**
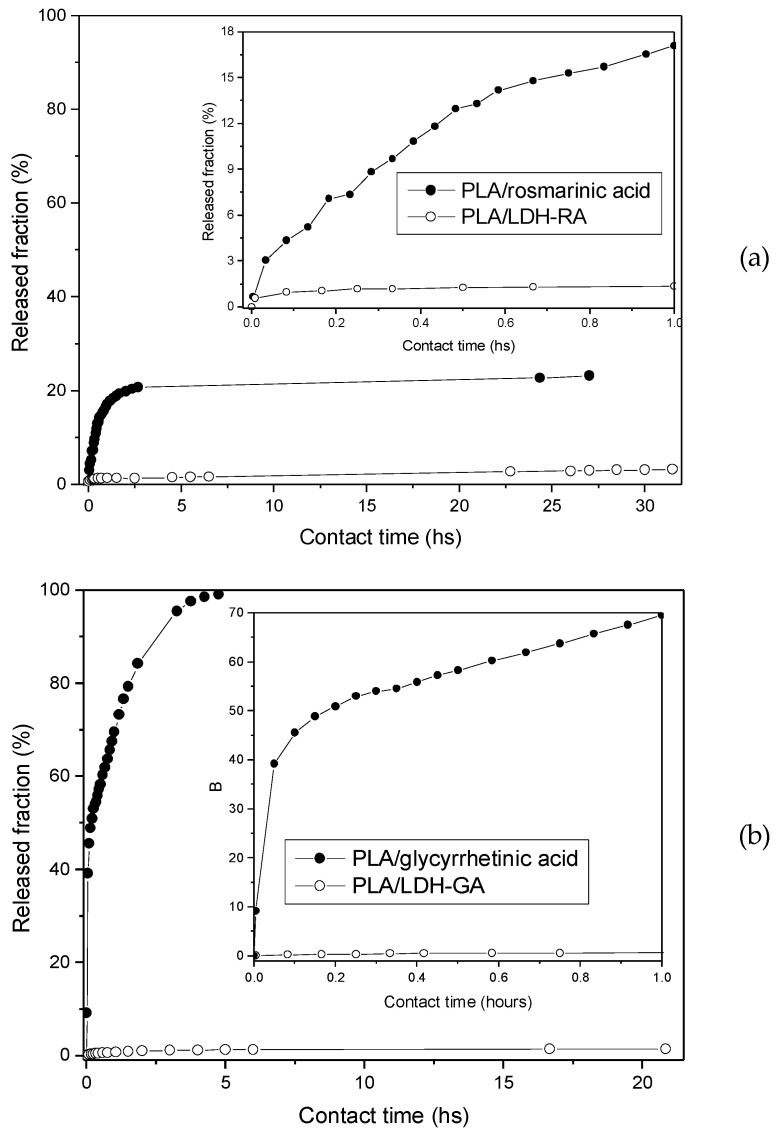
Released fraction of active molecules from PLA/rosmarinic acid and PLA/LDH-RA films (**a**) and from PLA/glycyrrhetinic acid and PLA/LDH-GA films (**b**) as a function of contact time. The inset shows the curves for the first hour of contact between the film and the EtOH/H_2_O (95/5 *v*/*v*) solution.

For comparison, rosmarinic and glycyrrhetinic acid migration was also performed from PLA film containing the free acids dispersed in the matrix in the same amount contained in PLA/LDH-RA and PLA/LDH-GA. This experiment is interesting to assess whether the intercalation of active molecules into LDH affects their migration. The released fractions were calculated as the percentage of the released amount of active molecules to the total amount in the films and are reported as a function of contact time (Figure 11).

Data reported in Figure 11 show the fast release of the free rosmarinic and glycyrrhetinic acids from the PLA film, where the two molecules are mixed and not immobilized in the LDH. This effect is particularly evident in the case of PLA/glycyrrhetinic acid, as about 70% of the acid migrates from the PLA film after 1 h, and about 40% migrates in 10 min. Interestingly, the migration of the active molecules from PLA/LDH-RA and PLA/LDH-GA composites was instead very limited, and only after 20–30 h of contact the released fraction reached about 3% in the case of PLA/LDH-RA and less than 2% in the case of PLA/LDH-GA. These data confirmed that immobilization by the intercalation/absorption of GA and RA in LDH effectively controls their migration from PLA, ensuring the prolonged functional activity of the PLA composites over time.

#### 2.2.4. Antioxidant Capacity

The antioxidant activity of pure compounds and plant extracts in vitro and in vivo is generally tested using the DPPH assay. Therefore, we applied this method to test the antioxidant properties of RA, LDH-RA, and PLA/LDH-RA films. For comparison, Trolox, a water-soluble vitamin E analogue, was used as a reference. Between the different experimental conditions reported in the literature, we chose to follow the reaction until no more variations in the absorbance of DPPH at 515 nm occurred [66]. Furthermore, from a preliminary analysis, we set the end of the test at 24 h; indeed, the degradation of DPPH is limited, and the reaction between DPPH and the antioxidant is complete. To report the antioxidant activity, we used the EC_50_ value, which is the effective antioxidant concentration necessary to decrease the initial DPPH concentration by 50%. A lower value of EC_50_ is associated with higher antioxidant activity. The EC_50_ is graphically evaluated by reporting the reduction percentage of the DPPH solution (I%), i.e., the percentage of inhibition or quenching (Equation (1) in the Supplementary Materials), as a function of the antioxidant concentration. For concentrations near the EC_50_ value, the correlation between I% and the antioxidant concentration is linear, and a linear fitting of the experimental data can be used to evaluate the EC_50_.

To favor the delamination of layered LDH and to increase the quantity of available RA molecules, a methanol suspension of LDH-RA was probe-sonicated for 10 min before the analysis. Then, different aliquots of this suspension were added to the DPPH solution. To evaluate the EC_50_ of LDH-RA, we considered the theoretical molar concentration of RA available in LDH-RA (37 wt.%) and LDH-DS/RA (13 wt.%), as reported in Paragraph 2.1. Table 2 shows the EC_50_ values of rosmarinic acid, LDH-RA, and LDH-DS/RA compared with that of Trolox.

The EC_50_ value of Trolox is in good agreement with the literature data [67]. The data in Table 2 show that rosmarinic acid, LDH-RA, and LDH-DS/RA have a higher antioxidant ability than Trolox since a lower concentration of rosmarinic acid, LDH-RA, or LDH-DS/RA is necessary to decrease the initial DPPH concentration by 50%. Their activity is even higher than other common antioxidants frequently added to polymer packaging, such as 2,6-di-tert-butyl-4-methyl phenyl (BHT), whose EC_50_ is equal to 18.9 μM [66]. Furthermore, the antioxidant activity of LDH-RA and LDH-DS/RA is lower than that of rosmarinic acid.

In the AP design, besides the need for the controlled migration of antioxidants from polymer films, it is necessary that the released molecules and polymer films maintain their antioxidant properties. In agreement with this observation, we carried out the DPPH test on the ethanol solution resulting from the migration of RA from the PLA/LDH-RA film. In this case, the reduction percentage of the DPPH solution (I%) (Equation (1) in the Supplementary Materials) was 85%. This result shows that most of the DPPH reacted with RA, confirming that leached RA maintains its antioxidant ability. As for the demonstration of the polymer film’s antioxidant activity, in agreement with procedures reported in the literature [68,69], the PLA/LDH-RA film was suspended in a DPPH solution, and the decrease in the DPPH signal at 515 nm was followed by UV-vis spectroscopy (Figure S8). From the graph reported in Figure S8, it is evident that the film also has an antioxidant ability and that the EC_50_ value is reached in about 3 h. Finally, at the steady state, the reduction percentage of the DPPH solution was more than 90%.

#### 2.2.5. Antibacterial Activity

The antibacterial activity of films of PLA/LDH-RA and PLA/LDH-GA, prepared by melt mixing (see Supplementary Materials), was evaluated against two pathogens: Gram-negative *Escherichia coli* and Gram-positive *Staphylococcus aureus*, which are standard indicators of food contamination in the food industry and are capable of adhering, colonizing, and forming biofilms on surfaces. For the test, we followed the standard method ISO 22196:2011, composed of the steps described in the experimental section (Figure S9). The comparison of the number of bacteria that are present immediately after their inoculation in the test specimen and after a fixed time gives a percentage index (antibacterial activity R), which determines the effectiveness of an antibacterial agent or treatment (Table 3).

The surface of the PLA/LDH-RA sample shows R ≥ 2 logs CFU/cm^2^ for both bacteria. Although ISO 22196:2011 reports that the criteria for the determining antimicrobial effectiveness are case-specific, the Japanese standard (JIS Z 2801) derived from ISO 22196 states that to be significant, the R-value must be at least 2.0. So, based on the Japanese standard, LDH-RA is highly inhibitory to both *E. coli* and *S. aureus*. Therefore, the PLA/LDH-RA sample may be used as an effective contact-active antimicrobial polymer. However, PLA/LDH-GA shows only the weak inhibition of both bacteria. In the first instance, this different behavior could be due to differences in the activity of RA and GA towards the two bacteria species. Nonetheless, even though the ISO 22196 method is a contact test, the migration of RA and GA from LDH lamellae embedded into the polymer matrix could influence the result. Furthermore, the possible different morphologies of the composites and the distribution in the bulk and surface of the modified LDHs could also influence the results of the antimicrobial tests. For this reason, further tests are necessary to clarify the antimicrobial activity of these materials and how the release kinetics of active molecules can affect these tests. In addition, different bacterial strains should be tested. However, the results obtained in this preliminary study strongly support the effectiveness of this method for the preparation of AP.

## 3. Materials and Methods

### 3.1. Materials

LDH intercalated with the nitrate anion (LDH-NO_3_) with the molecular formula [Mg_0.66_Al_0.34_(OH)_2_](NO_3_)_0.34_·0.5 H_2_O was purchased from Prolabin & Tefarm (Perugia, Italy). LDH modified with sodium dodecyl sulfate (LDH-DS) prepared as reported in the literature [53] was used for comparison purposes. Rosmarinic acid 96%, 18β-glycyrrhetinic acid 97%, sodium dodecyl sulfate (NaDS) ≥ 99.0%, 2,2-diphenyl-1-picrylhydrazyl hydrate (DPPH), and 6-hydroxy-2,5,7,8-tetramethylchroman-2-carboxylic acid (Trolox) 97%, all purchased from Sigma-Aldrich (St. Louis, MO, USA), were used as received. IR-grade KBr (Pike Technologies) was used as received.

Poly(lactic acid) (PLA) Ingeo TM 2003D, Extrusion Grade, with a density of 1.24 g cm^−3^, 4.1 wt% D isomer (NatureWorks LLC, Plymouth, MN, USA), and poly(butylene succinate) (PBS) BioPBS FD92PM with a density of 1.24 g cm^−3^, produced by Mitsubishi Chemical Corporation (Tokyo, Japan), were used as polymer matrices. Chloroform ACS reagent ≥ 99.8%, chloroform, HPLC grade, ≥99.8% (ethanol-stabilized), and ethanol ACS reagent 96% from Sigma-Aldrich and acetone ACS reagent ≥ 99.6% and methanol 99+% extra pure from Acros Organics (Geel, Belgium) were used as received.

All suspensions were prepared with CO_2_-free deionized ultrapure water (18.2 MOhm cm) obtained using a Milli-Q system (Millipore, Bedford, MA, USA).

### 3.2. Preparation of Modified LDHs (LDH-RA, LDH-GA, LDH-DS/RA, and LDH-DS/GA)

Anion exchange was carried out by using the quantities reported in Table 4. All preparation was carried out under nitrogen using CO_2_-free deionized water and, when necessary, ethanol as a solvent. After mixing LDH-NO_3_ and intercalating agents, the pH of the suspension was about 6–7. Stirring was carried out for 48 h. The product was then recovered by filtration, followed by repeated washing with CO_2_-free deionized water. The product was then dried under vacuum at 40 °C for 24 h.

### 3.3. Preparation of Polymer Composites

Polymer/LDH samples, containing 5 wt.% of modified LDH with respect to the polymer matrix, were prepared by solution mixing. In a typical experiment, modified LDH (0.1 g) was suspended in ethanol (5 mL), stirred, and probe-sonicated for 30 min using a Hielscher Ultrasonic Processor UP200St (200 W, 26 kHz, Hielscher Ultrasonics, Teltow, Germany) equipped with a titanium 2 mm sonotrode (S26d2), which was used at 50% of the maximum amplitude at an effective power density of 4 W·cm^−2^. The modified-LDH suspension was then added dropwise to a polymer–chloroform solution (2 g of PLA, PBS, or PLA/PBS 60/40 wt./wt. mixture in 30 mL of CHCl_3_) and kept under stirring for 2 h at room temperature. Finally, the solvent was removed by evaporation under vacuum, and composites were dried under vacuum at 50 °C for 24 h.

PLA, PBS, and PLA/PBS (60/40 wt./wt.) matrix composites were prepared for each modified LDH. Finally, thin films of the composites were obtained by compression molding using a Carver 3851-0 press, setting the temperature of the plates to of 180 °C and applying a pressure equal to 4 bar for a duration of 2 min (using an amount of composite of about 0.5 g). Squares of 5 cm per side were then cut from the thin films (about 100 μm thickness), which were used for all characterizations.

### 3.4. Characterization

X-ray diffraction (XRD) analysis was performed at room temperature with an X’Pert PRO (Malvern Panalytical Company, Malvern, UK) powder diffractometer using Cu Kα radiation (1.541874 Å), a nickel filter with a thickness of 0.02 nm, and a fast detector (PIXcel) with an active range of 3.347°. Spectra were acquired in the 2θ range from 1.5 to 30°, applying a step size of 0.0131° and a counting time of 207.5 s. The basal distance of the LDH was computed from the diffraction signal (003) by applying Bragg’s law (Table 1). LDH samples were characterized as powders. Polymer-based nanocomposites were analyzed as films prepared by compression molding and annealed at 80 °C for 6 h.

Infrared spectra (FT-IR) were recorded with a Fourier Transform Spectrometer (Perkin Elmer Spectrum Two; Perkin-Elmer, Waltham, MA, USA) in the 400–4000 cm^−1^ range with a resolution of 4 cm^−1^ using 16 scans. The spectra of LDHs and of the organic anions were obtained by mixing the samples with potassium bromide. The spectra of polymer-based composites were registered on thin films obtained by solution casting on the KBr window of a chloroform solution prepared by dissolving 5 mg of sample in 1 mL of chloroform. The spectra were processed with the instrument software Spectrum.

UV-vis absorption spectra were recorded at room temperature with a Jasco V-750 UV-visible spectrophotometer (Jasco International Co. Ltd., Tokyo, Japan). Calibration curves of RA and GA were obtained by recording the absorbance of solutions of each molecule at different concentrations and by performing a linear fitting of the absorbance as a function of the molar concentration of the molecules (Figure S4). The absorbance was recorded at the maximum wavelength for each molecule, which was 330 nm for RA and 250 nm for GA. The following molar extinction coefficients were calculated: RA ε_330_ = 18,500 M^−1^ cm^−1^ and GA ε_250_ = 10,800 M^−1^ cm^−1^.

Determinations of organic acid content in modified LDHs were carried out by dissolving a known amount (2–3 mg) of each LDH in concentrated HCl (a few drops) and diluting with EtOH to obtain solutions having an absorbance lower than 1. UV-vis spectra of the solutions were recorded and, based on molar extinction coefficients previously reported, the amount of the organophilic fraction was determined (Table 1).

Thermogravimetric analysis (TGA) was performed using an SII TG/DTA 7200 EXSTAR (Seiko instrument, Chiba, Japan). LDH samples (5–10 mg) were placed in alumina sample pans (70 μL), and runs were carried out at the standard rate of 10 °C min^−1^ from 30 to 900 °C under air flow (200 mL min^−1^). In the case of PLA, PBS, and PLA/PBS nanocomposites, samples (5–10 mg) were placed in alumina sample pans (70 μL), and runs were carried out at the standard rate of 10 °C min^−1^ from 30 to 600 °C under nitrogen and from 600 to 900 °C under air flow (200 mL min^−1^).

Differential Scanning Calorimetry (DSC) measurements were performed on 5–10 mg samples, placed in aluminum pans, under a nitrogen atmosphere (nitrogen flow was 50 mL min^−1^ for all the experiments) by using a Perkin-Elmer DSC 4000 differential scanning calorimeter thermal analyzer (Perkin-Elmer, Waltham, MA, USA) equipped with a 3-stage cooler able to reach −130 °C. Previously, the instrument was calibrated by using indium (m. p. 156.6 °C, ΔH_m_ = 28.5 J g^−1^) and zinc (m. p. 419.5 °C) as references. The polymer-based nanocomposites were subjected to the following thermal cycle: first heating from 30 to 180 °C at 10 °C/min, keeping the temperature at 180 °C for 3 min, cooling from 180 to −70 °C at 10 °C/min, keeping the temperature at −70 °C for 3 min, and second heating from −70 to 180 °C at 10 °C/min. Crystallization and melting temperatures were evaluated from the second heating, and the relative enthalpies were calculated from the integrated areas of crystallization and melting peaks, using the instrument software Pyris V9.0.

Size exclusion chromatography (SEC) analysis of the polymers was performed in CHCl_3_ at a flow rate of 0.3 mL min^−1^ using an Agilent Technologies 1260 Series instrument (Agilent Technologies, Santa Clara, CA, USA) equipped with a degasser, an isocratic HPLC pump, a refractive index (RI) detector, and one pre-column PLgel 5 μm and two PLgel MiniMIX-D 5 μm columns conditioned at 35 °C. The system was calibrated with polystyrene standards in a range from 500 to 3 × 10^5^ g mol^−1^. Samples were dissolved in CHCl_3_ (3 mg ml^−1^) and filtered through a 0.20 μm syringe filter before analysis. The number average molecular weight (M_n_) and weight average molecular weight (M_w_) were calculated using the Agilent ChemStation software.

The morphological analysis was performed by using a Field-Emission Scanning Electron Microscope, FE-SEM, with an FEI Quanta 450 ESEM FEG instrument in SE mode located at CISUP (Centre for Instrumentation Sharing—University of Pisa). The cryo-fractured surface of the sample was covered with an ultrathin gold layer prior to imaging.

Descriptions of migration tests and of the methods used to evaluate the antioxidant and antibacterial activities are reported in the Supplementary Materials.

## 4. Conclusions

Developing composites consisting of biodegradable polymeric matrices derived from renewable sources and endowed with antioxidant and antimicrobial properties is crucial for advancing new industrially relevant materials for application in AP. We have demonstrated the possibility of immobilizing naturally derived bioactive molecules, such as rosmarinic and glycyrrhetinic acids in their mono-deprotonated forms, on nanostructured inorganic substrates such as LDH through an anion-exchange process conducted in water. Rosmarinate and glycyrrhetinate anions have been intercalated into LDH alone or with an alkyl surfactant. The dispersion of these host–guest systems in PLA, PBS, and a PLA/PBS blend, the latter selected because it has more suitable mechanical properties for possible application in flexible packaging, was accomplished by solution mixing and subsequent die-casting film formation. Morphological analyses showed a better dispersion in the polymer matrices of the systems co-intercalated with the surfactant than those modified with the rosmarinate or glycyrrhetinate anions alone. The co-intercalated fillers also promoted a compatibilizing action on the PLA/PBS blend, as confirmed by the thermal investigation. We believe that the improved interphase compatibility compared with the pure blend is related to the arrangement of the lamellae at the interface between the phases, resulting in a reduction in the size of the dispersed phase.

We also demonstrated that the active species migrate slowly from the thin films of the composite materials in a hydroalcoholic solvent, confirming the control of the release process. Furthermore, we verified that the intercalated rosmarinate retains the antioxidant capacity of the free molecule and that this property is also transferred to a PLA film containing the dispersed filler. Finally, for two PLA composites selected and produced by a process in the melt and containing LDH-RA and LDH-GA, the inhibitory capacity against *E. coli* and *S. aureus* was evaluated using in vitro contact tests on thin films, which showed that the PLA/LDH-RA sample possesses complete antimicrobial activity against the two selected species. In contrast, the PLA/LDH-GA sample shows weak inhibition against both bacteria.

## Figures and Tables

**Table 1:** Basal distance, water loss, TGA residue, and quantity of active molecules for modified LDHs.

Modified LDH	Basal Distance ^1^ (nm)	Water ^2^ (wt. %)	TGA Residue (wt. %)	Active Molecule Content ^3^ (wt. %)
LDH-RA	2.26	5.5	29.0	37
LDH-GA	2.85	10.7	35.0	45
LDH-DS/RA	2.52	8.0	33.1	13
LDH-DS/GA	2.85	7.7	44.3	13

^1^ The basal distance of LDH-NO_3_ is 0.89 nm [53] and the basal distance of LDH-DS is 2.52 nm [52]. ^2^ The quantity of water (adsorbed and co-intercalated) is given as a percentage by weight of the hybrid, and it has been calculated as the TGA weight loss between 30 and 150–200 °C. ^3^ The amount of active molecule (RA or GA anions) has been evaluated via UV-vis spectroscopy by dissolving a known amount of modified LDH in concentrated HCl solution.

**Table 2:** EC_50_ values of Trolox, rosmarinic acid, LDH-RA, and LDH-DS/RA.

Sample	EC_50_ (μM)
Trolox	14.2 ± 0.3
Rosmarinic acid	1.74 ± 0.04
LDH-RA	8.3 ± 0.17
LDH-DS/RA	7.0 ± 0.5

**Table 3:** Antibacterial activity (R) of PLA/LDH-RA and PLA/LDH-GA against *E. coli* and *S. aureus*.

Sample	Antibacterial Activity (R) (Log CFU/cm^2^)	
	*E. coli*	*S. aureus*
PLA/LDH-RA	2.56	2.74
PLA/LDH-GA	0.20	0.62

**Table 4:** Quantities of LDH-NO_3_, rosmarinic acid, mono-sodium salt of glycyrrhetinic acid, and NaDS used for the preparation of modified LDHs.

Modified LDH	LDH-NO_3_ (g)	Intercalating Anion (g) [mmol]	NaDS (g) [mmol]	Solvent (mL)	Yield (g)
LDH-RA	1.0	RA (1.37) [2.91]	-	H_2_O (50)	1.37
LDH-GA ^1^	0.9	NaGA ^2^ (1.62) [3.29]	-	H_2_O/EtOH 40/60 (50)	1.31
LDH-DS/RA	0.73	RA (0.50) [1.39]	NaDS (0.4) [1.39]	H_2_O (50)	1.03
LDH-DS/GA	0.73	GA (0.65) [1.39]	NaDS (0.4) [1.39]	H_2_O/EtOH 80/20 (50)	0.86

^1^ The anion-exchange reaction was carried out at 55 °C. ^2^ NaGA was prepared according to the method of Wu et al. [49].

## Data Availability

Data are contained within the article or Supplementary Materials.

## Supplementary Materials

The following supporting information can be downloaded at: https://www.mdpi.com/article/10.3390/molecules28010347/s1. Figure S1: FT-IR spectra of NaRA, NaGA, and LDH-DS; Figure S2: TGA and DTG curves of samples LDH-RA (a), LDH-GA (b), LDH-DS/RA (c), and LDH-DS/GA (d); Figure S3: TGA and DTG curves of rosmarinic acid, NaGA, LDH-NO_3_, and LDH-DS; Figure S4. UV-vis spectra of rosmarinic acid ethanol solutions at different concentrations and calibration curve (a) and UV-vis spectra of glycyrrhetinic acid ethanol solutions at different concentrations and calibration curve (b); Figure S5: SEM images at different magnifications of PLA/LDH-RA (a and a’) and PLA/LDH-GA (b and b’); Figure S6: DSC curves of PLA (black), PBS (red), and PLA/PBS blend (green) (second heating). Red and black dotted lines indicate the fundamental transitions of pure PBS and PLA, respectively; Figure S7: DSC curves (cooling) of PLA/PBS blend and its composites. Black dotted lines indicate the fundamental transitions of PLA/PBS blend; Figure S8: I% as a function of time for PLA/LDH-RA film in contact with DPPH stock solution; Figure S9: Schematic representation of antibacterial test by standard method ISO 22196:2011 with pictures registered during the experiment. Table S1: Molecular weights and distributions of composites and their blanks; Table S2: DSC results for PLA, PBS, PLA/PBS, and their composites. Description of migration tests and of the methods used to evaluate the antioxidant and antibacterial activities.

## Sample Availability

Samples are available from the authors.

## References

[1] Rasal R.M., Janorkar A.V., Hirt D.E. (2010). Poly(Lactic Acid) Modifications. Prog. Polym. Sci..

[2] Arrieta M.P., Fortunati E., Dominici F., López J., Kenny J.M. (2015). Bionanocomposite Films Based on Plasticized PLA-PHB/Cellulose Nanocrystal Blends. Carbohydr. Polym..

[3] Murariu M., Dechief A.L., Ramy-Ratiarison R., Paint Y., Raquez J.M., Dubois P. (2015). Recent Advances in Production of Poly(Lactic Acid) (PLA) Nanocomposites: A Versatile Method to Tune Crystallization Properties of PLA. Nanocomposites.

[4] Ncube L.K., Ude A.U., Ogunmuyiwa E.N., Zulkifli R., Beas I.N. (2020). Environmental Impact of Food Packaging Materials: A Review of Contemporary Development from Conventional Plastics to Polylactic Acid Based Materials. Materials.

[5] Pivsa-Art W., Pavasupree S., O-Charoen N., Insuan U., Jailak P., Pivsa-Art S. (2011). Preparation of Polymer Blends between Poly (L-Lactic Acid), Poly (Butylene Succinate-Co-Adipate) and Poly (Butylene Adipate-Co-Terephthalate) for Blow Film Industrial Application. Energy Procedia.

[6] Gigante V., Coltelli M.B., Vannozzi A., Panariello L., Fusco A., Trombi L., Donnarumma G., Danti S., Lazzeri A. (2019). Flat Die Extruded Biocompatible Poly(Lactic Acid) (PLA)/Poly(Butylene Succinate) (PBS) Based Films. Polymers.

[7] Arrieta M.P., Samper M.D., Aldas M., López J. (2017). On the Use of PLA-PHB Blends for Sustainable Food Packaging Applications. Materials.

[8] Platnieks O., Gaidukovs S., Thakur V.K., Barkane A., Beluns S. (2021). Bio-Based Poly (Butylene Succinate): Recent Progress, Challenges and Future Opportunities. Eur. Polym. J..

[9] Su S., Kopitzky R., Tolga S., Kabasci S. (2019). Polylactide (PLA) and Its Blends with Poly(Butylene Succinate) (PBS): A Brief Review. Polymers.

[10] Barletta M., Aversa C., Ayyoob M., Gisario A., Hamad K., Mehrpouya M., Vahabi H. (2022). Poly(Butylene Succinate) (PBS): Materials, Processing, and Industrial Applications. Prog. Polym. Sci..

[11] Suwanamornlert P., Kerddonfag N., Sane A., Chinsirikul W., Zhou W., Chonhenchob V. (2020). Poly(Lactic Acid)/Poly(Butylene-Succinate-Co-Adipate) (PLA/PBSA) Blend Films Containing Thymol as Alternative to Synthetic Preservatives for Active Packaging of Bread. Food Packag. Shelf Life.

[12] Qin Y., Liu D., Wu Y., Yuan M., Li L., Yang J. (2015). Effect of PLA/PCL/Cinnamaldehyde Antimicrobial Packaging on Physicochemical and Microbial Quality of Button Mushroom (*Agaricus Bisporus*). Postharvest Biol. Technol..

[13] Yildirim S., Röcker B., Pettersen M.K., Nilsen-Nygaard J., Ayhan Z., Rutkaite R., Radusin T., Suminska P., Marcos B., Coma V. (2018). Active Packaging Applications for Food. Compr. Rev. Food Sci. Food Saf..

[14] Quintavalla S., Vicini L. (2002). Antimicrobial Food Packaging in Meat Industry. Meat Ind..

[15] Dutta D., Sit N. (2022). Application of Natural Extracts as Active Ingredient in Biopolymer Based Packaging Systems. J. Food Sci. Technol..

[16] Bahrami A., Delshadi R., Assadpour E., Jafari S.M., Williams L. (2020). Antimicrobial-Loaded Nanocarriers for Food Packaging Applications. Adv. Colloid. Interface Sci..

[17] Gorrasi G., Sorrentino A. (2019). Layered Double Hydroxide Polymer Nanocomposites for Food-Packaging Applications. Layered Double Hydroxide Polymer Nanocomposites.

[18] Mishra G., Dash B., Pandey S. (2018). Layered Double Hydroxides: A Brief Review from Fundamentals to Application as Evolving Biomaterials. Appl. Clay Sci..

[19] Mochane M.J., Magagula S.I., Sefadi J.S., Sadiku E.R., Mokhena T.C. (2020). Morphology, Thermal Stability, and Flammability Properties of Polymer-Layered Double Hydroxide (LDH) Nanocomposites: A Review. Crystals.

[20] Costantino U., Ambrogi V., Nocchetti M., Perioli L. (2008). Hydrotalcite-like Compounds: Versatile Layered Hosts of Molecular Anions with Biological Activity. Microporous Mesoporous Mater..

[21] Gorrasi G., Bugatti V., Vertuccio L., Vittoria V., Pace B., Cefola M., Quintieri L., Bernardo P., Clarizia G. (2020). Active Packaging for Table Grapes: Evaluation of Antimicrobial Performances of Packaging for Shelf Life of the Grapes under Thermal Stress. Food Packag. Shelf Life.

[22] Bugatti V., Vertuccio L., Zuppardi F., Vittoria V., Gorrasi G. (2019). Pet and Active Coating Based on a Ldh Nanofiller Hosting P-Hydroxybenzoate and Food-Grade Zeolites: Evaluation of Antimicrobial Activity of Packaging and Shelf Life of Red Meat. Nanomaterials.

[23] Pérez Amaro L., Cicogna F., Passaglia E., Morici E., Oberhauser W., Al-Malaika S., Dintcheva N.T., Coiai S. (2016). Thermo-Oxidative Stabilization of Poly(Lactic Acid) with Antioxidant Intercalated Layered Double Hydroxides. Polym. Degrad. Stab..

[24] Coiai S., Cicogna F., Pinna S., Spiniello R., Onor M., Oberhauser W., Coltelli M.B., Passaglia E. (2021). Antibacterial LDPE-Based Nanocomposites with Salicylic and Rosmarinic Acid-Modified Layered Double Hydroxides. Appl. Clay Sci..

[25] Jayakumar A., Radoor S., Kim J.T., Rhim J.W., Nandi D., Parameswaranpillai J., Siengchin S. (2022). Recent Innovations in Bionanocomposites-Based Food Packaging Films—A Comprehensive Review. Food Packag. Shelf Life.

[26] Glaskova-Kuzmina T., Starkova O., Gaidukovs S., Platnieks O., Gaidukova G. (2021). Durability of Biodegradable Polymer Nanocomposites. Polymers.

[27] Pisoschi A.M., Pop A., Georgescu C., Turcuş V., Olah N.K., Mathe E. (2018). An Overview of Natural Antimicrobials Role in Food. Eur. J. Med. Chem..

[28] Gyawali R., Ibrahim S.A. (2014). Natural Products as Antimicrobial Agents. Food Control.

[29] Coban H.B. (2020). Organic Acids as Antimicrobial Food Agents: Applications and Microbial Productions. Bioprocess Biosyst. Eng..

[30] Plumridge A., Stratford M., Lowe K.C., Archer D.B. (2008). The Weak-Acid Preservative Sorbic Acid Is Decarboxylated and Detoxified by a Phenylacrylic Acid Decarboxylase, PadA1, in the Spoilage Mold Aspergillus Niger. Appl. Environ. Microbiol..

[31] Ouattara B., Simard R.E., Piette G., Begin A., Holley R.A. (2000). Inhibition of Surface Spoilage Bacteria in Processed Meats by Application of Antimicrobial Films Prepared with Chitosan. Int. J. Food Microbiol..

[32] Daglia M. (2012). Polyphenols as Antimicrobial Agents. Curr. Opin. Biotechnol..

[33] Radovanović B.C., Andelković A.S.M., Radovanović A.B., Andelković M.Z. (2013). Antioxidant and Antimicrobial Activity of Polyphenol Extracts from Wild Berry Fruits Grown in Southeast Serbia. Trop. J. Pharm. Res..

[34] Prabakaran M., Kim S.H., Sasireka A., Chandrasekaran M., Chung I.M. (2018). Polyphenol Composition and Antimicrobial Activity of Various Solvent Extracts from Different Plant Parts of Moringa Oleifera. Food Biosci..

[35] Coiai S., Campanella B., Paulert R., Cicogna F., Bramanti E., Lazzeri A., Pistelli L., Coltelli M.B. (2021). Rosmarinic Acid and Ulvan from Terrestrial and Marine Sources in Anti-Microbial Bionanosystems and Biomaterials. Appl. Sci..

[36] Bais H.P., Walker T.S., Schweizer H.P., Vivanco J.M. (2002). Root Specific Elicitation and Antimicrobial Activity of Rosmarinic Acid in Hairy Root Cultures of Ocimum Basilicum. Plant Physiol. Biochem..

[37] Kim G.D., Park Y.S., Jin Y.H., Park C.S. (2015). Production and Applications of Rosmarinic Acid and Structurally Related Compounds. Appl. Microbiol. Biotechnol..

[38] Kowalska A., Kalinowska-Lis U. (2019). 18β-Glycyrrhetinic Acid: Its Core Biological Properties and Dermatological Applications. Int. J. Cosmet. Sci..

[39] Wang L., Yang R., Yuan B., Liu Y., Liu C. (2015). The Antiviral and Antimicrobial Activities of Licorice, a Widely-Used Chinese Herb. Acta Pharm. Sin. B.

[40] Kim J.K., Oh S.M., Kwon H.S., Oh Y.S., Lim S.S., Shin H.K. (2006). Anti-Inflammatory Effect of Roasted Licorice Extracts on Lipopolysaccharide-Induced Inflammatory Responses in Murine Macrophages. Biochem. Biophys. Res. Commun..

[41] Zhou J.X., Wink M. (2019). Evidence for Anti-Inflammatory Activity of Isoliquiritigenin, 18β Glycyrrhetinic Acid, Ursolic Acid, and the Traditional Chinese Medicine Plants Glycyrrhiza Glabra and Eriobotrya Japonica, at the Molecular Level. Medicines.

[42] Oyama K., Kawada-Matsuo M., Oogai Y., Hayashi T., Nakamura N., Komatsuzawa H. (2016). Antibacterial Effects of Glycyrrhetinic Acid and Its Derivatives on Staphylococcus Aureus. PLoS ONE.

[43] Huang L.R., Hao X.J., Li Q.J., Wang D.P., Zhang J.X., Luo H., Yang X.S. (2016). 18β-Glycyrrhetinic Acid Derivatives Possessing a Trihydroxylated a Ring Are Potent Gram-Positive Antibacterial Agents. J. Nat. Prod..

[44] Lagreca E., Onesto V., di Natale C., la Manna S., Netti P.A., Vecchione R. (2020). Recent Advances in the Formulation of PLGA Microparticles for Controlled Drug Delivery. Prog. Biomater..

[45] Zeeshan M., Ali H., Khan S., Mukhtar M., Khan M.I., Arshad M. (2019). Glycyrrhizic Acid-Loaded PH-Sensitive Poly-(Lactic-Co-Glycolic Acid) Nanoparticles for the Amelioration of Inflammatory Bowel Disease. Nanomedicine.

[46] Pan X., Liu S., Ju L., Xi J., He R., Zhao Y., Zhuang R., Huang J. (2020). Preparation, Evaluation, and in Vitro Cytotoxicity Studies of Artesunate-Loaded Glycyrrhetinic Acid Decorated PEG-PLGA Nanoparticles. Drug. Dev. Ind. Pharm..

[47] Darvishi B., Manoochehri S., Kamalinia G., Samadi N., Amini M., Mostafavi S.H., Maghazei S., Atyabi F., Dinarvand R. (2015). Preparation and Antibacterial Activity Evaluation of 18-β-Glycyrrhetinic Acid Loaded PLGA Nanoparticles. Iran J. Pharm. Res..

[48] Bag B.G., Majumdar R. (2012). Self-Assembly of a Renewable Nano-Sized Triterpenoid 18β-Glycyrrhetinic Acid. RSC Adv..

[49] Wu J., Lu J., Hu J., Gao Y., Ma Q., Ju Y. (2013). Self-Assembly of Sodium Glycyrrhetinate into a Hydrogel: Characterisation and Properties. RSC Adv..

[50] Danti S., Trombi L., Fusco A., Azimi B., Lazzeri A., Morganti P., Coltelli M.B., Donnarumma G. (2019). Chitin Nanofibrils and Nanolignin as Functional Agents in Skin Regeneration. Int. J. Mol. Sci..

[51] Miletić A., Ristić I., Coltelli M.B., Pilić B. (2020). Modification of PLA-Based Films by Grafting or Coating. J. Funct. Biomater..

[52] Coiai S., Javarone S., Cicogna F., Oberhauser W., Onor M., Pucci A., Minei P., Iasilli G., Passaglia E. (2018). Fluorescent LDPE and PLA Nanocomposites Containing Fluorescein-Modified Layered Double Hydroxides and Their ON/OFF Responsive Behavior towards Humidity. Eur. Polym. J..

[53] Muksing N., Magaraphan R., Coiai S., Passaglia E. (2011). Effect of Surfactant Alkyl Chain Length on the Dispersion, and Thermal and Dynamic Mechanical Properties of LDPE/Organo-LDH Composites. Express Polym. Lett..

[54] Meyn M., Beneke K., Lagaly G. (1990). Anion-Exchange Reactions of Layered Double Hydroxides. Inorg. Chem..

[55] Świsłocka R., Regulska E., Karpińska J., Świderski G., Lewandowski W. (2019). Molecular Structure and Antioxidant Properties of Alkali Metal Salts of Rosmarinic Acid. Experimental and DFT Studies. Molecules.

[56] Pavan P.C., de Gomes A.G., Valim J.B. (1998). Adsorption of Sodium Dodecyl Sulfate on Layered Double Hydroxides. Microporous Mesoporous Mater..

[57] Du L., Qu B. (2006). Structural Characterization and Thermal Oxidation Properties of LLDPE/MgAl-LDH Nanocomposites. J. Mater. Chem..

[58] Pagano C., Perioli L., Latterini L., Nocchetti M., Ceccarini M.R., Marani M., Ramella D., Ricci M. (2019). Folic Acid-Layered Double Hydroxides Hybrids in Skin Formulations: Technological, Photochemical and in Vitro Cytotoxicity on Human Keratinocytes and Fibroblasts. Appl. Clay Sci..

[59] De-Eknamkul W., Ellis B.E. (1984). Rosmarinic Acid Production and Growth Characteristics of Anchusa Officinalis Cell Suspension Cultures. Planta Med..

[60] Patil S.K., Salunkhe V.R., Mohite S.K. (2012). Development and validation of UV spectrophotometric method for estimation of glycyrrhetinic acid in hydro-alcoholic extract of glycyrrhiza glabra. Int. J. Pharm. Chem. Biol. Sci..

[61] Hassan E., Wei Y., Jiao H., Muhuo Y. (2013). Dynamic Mechanical Properties and Thermal Stability of Poly (Lactic Acid) and Poly (Butylene Succinate) Blends Composites. J. Fiber Bioeng. Inf..

[62] Delpouve N., Saiter-Fourcin A., Coiai S., Cicogna F., Spiniello R., Oberhauser W., Legnaioli S., Ishak R., Passaglia E. (2020). Effects of Organo-LDH Dispersion on Thermal Stability, Crystallinity and Mechanical Features of PLA. Polymer.

[63] Filippone G., Dintcheva N.T., la Mantia F.P., Acierno D. (2010). Using Organoclay to Promote Morphology Refinement and Co-Continuity in High-Density Polyethylene/Polyamide 6 Blends—Effect of Filler Content and Polymer Matrix Composition. Polymer.

[64] Filippone G., Dintcheva N.T., Acierno D., la Mantia F.P. (2008). The Role of Organoclay in Promoting Co-Continuous Morphology in High-Density Poly(Ethylene)/Poly(Amide) 6 Blends. Polymer.

[65] Aragón-Gutiérrez A., Rosa E., Gallur M., López D., Hernández-Muñoz P., Gavara R. (2021). Melt-Processed Bioactive Evoh Films Incorporated with Ferulic Acid. Polymers.

[66] Mishra K., Ojha H., Chaudhury N.K. (2012). Estimation of Antiradical Properties of Antioxidants Using DPPH- Assay: A Critical Review and Results. Food Chem..

[67] Villaño D., Fernández-Pachón M.S., Moyá M.L., Troncoso A.M., García-Parrilla M.C. (2007). Radical Scavenging Ability of Polyphenolic Compounds towards DPPH Free Radical. Talanta.

[68] Tran T.N., Mai B.T., Setti C., Athanassiou A. (2020). Transparent Bioplastic Derived from CO2-Based Polymer Functionalized with Oregano Waste Extract toward Active Food Packaging. ACS Appl. Mater. Interfaces.

[69] García-Arroyo P., Arrieta M.P., Garcia-Garcia D., Cuervo-Rodríguez R., Fombuena V., Mancheño M.J., Segura J.L. (2020). Plasticized Poly(Lactic Acid) Reinforced with Antioxidant Covalent Organic Frameworks (COFs) as Novel Nanofillers Designed for Non-Migrating Active Packaging Applications. Polymer.

