# Supplementary material for: Rosmarinic and Glycyrrhetinic Acid-Modified Layered Double Hydroxides as Functional Additives for Poly(Lactic Acid)/Poly(Butylene Succinate) Blends
Source: Molecules. 2023 Jan 1;28(1):347. doi: 10.3390/molecules28010347 (PMC9822188; doi:10.3390/molecules28010347)
Supplement: Supplementary file 1 [file molecules-28-00347-s001.zip › molecules-2094795-supplementary.pdf]

molecules

Supplementary Materials

Rosmarinic and glycyrrethinic acid-modified layered double hydroxides as functional additives for Poly(lactic acid)/Poly(butylene succinate) blends

Francesca Cicogna [1,*], Elisa Passaglia [1], Matilde Benedettini [1], Werner Oberhauser [2], Randa Ishak [3], Francesca Signori [3], Serena Coiai [1,*]

[1] Consiglio Nazionale delle Ricerche, Istituto di Chimica dei Composti OrganoMetallici (CNR-ICCOM), SS Pisa, Via Moruzzi 1, 56124 Pisa, Italy
[2] Consiglio Nazionale delle Ricerche, Istituto di Chimica dei Composti OrganoMetallici (CNR-ICCOM), Via Madonna del Piano 10, 50019 Sesto Fiorentino (FI), Italy
[3] Dipartimento di Ingegneria Civile e Industriale, Università di Pisa, Largo L. Lazzarino 1, 56122, Pisa, Italy
* Correspondence: serena.coiai@pi.iccom.cnr.it and francesca.cicogna@pi.iccom.cnr.it. Tel.: +39 050 315 2556 (SC); +39 050 315 3393(FC)

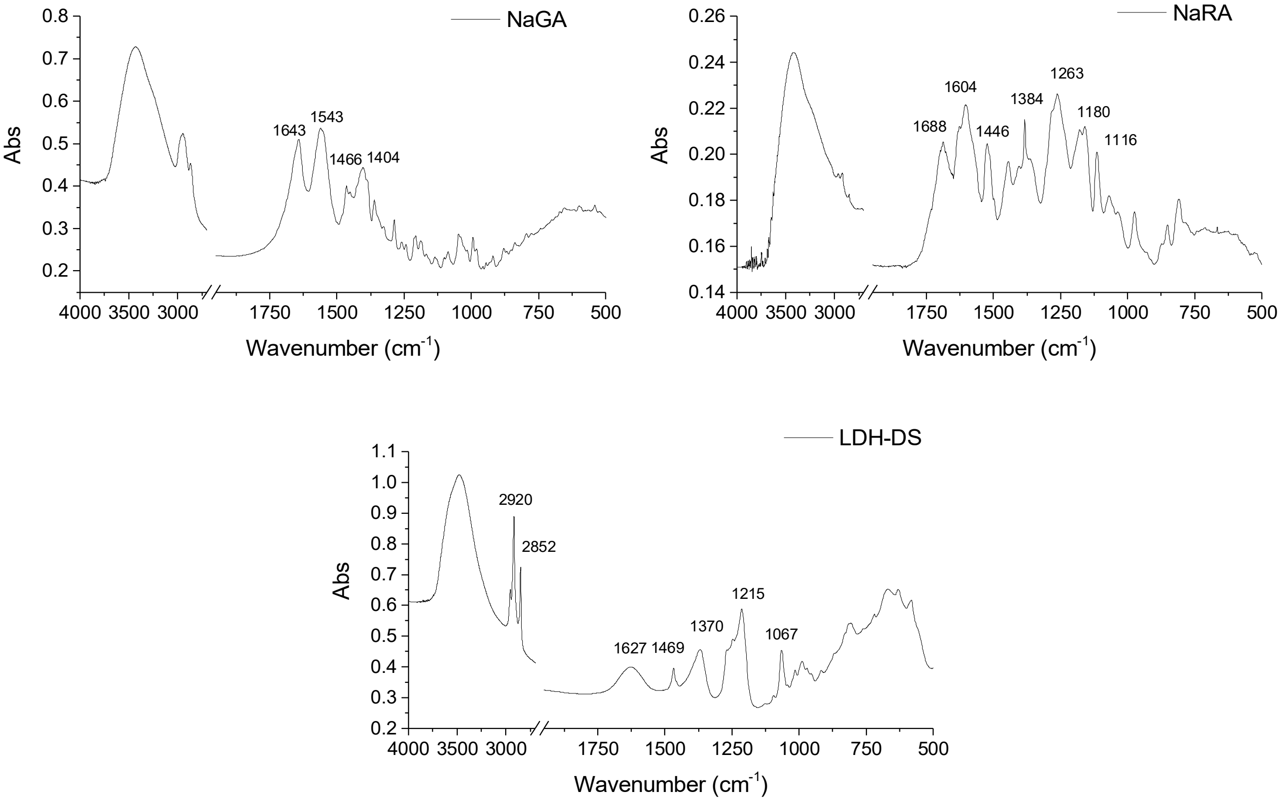

Figure S1. FT-IR spectra of NaRA, NaGA and LDH-DS

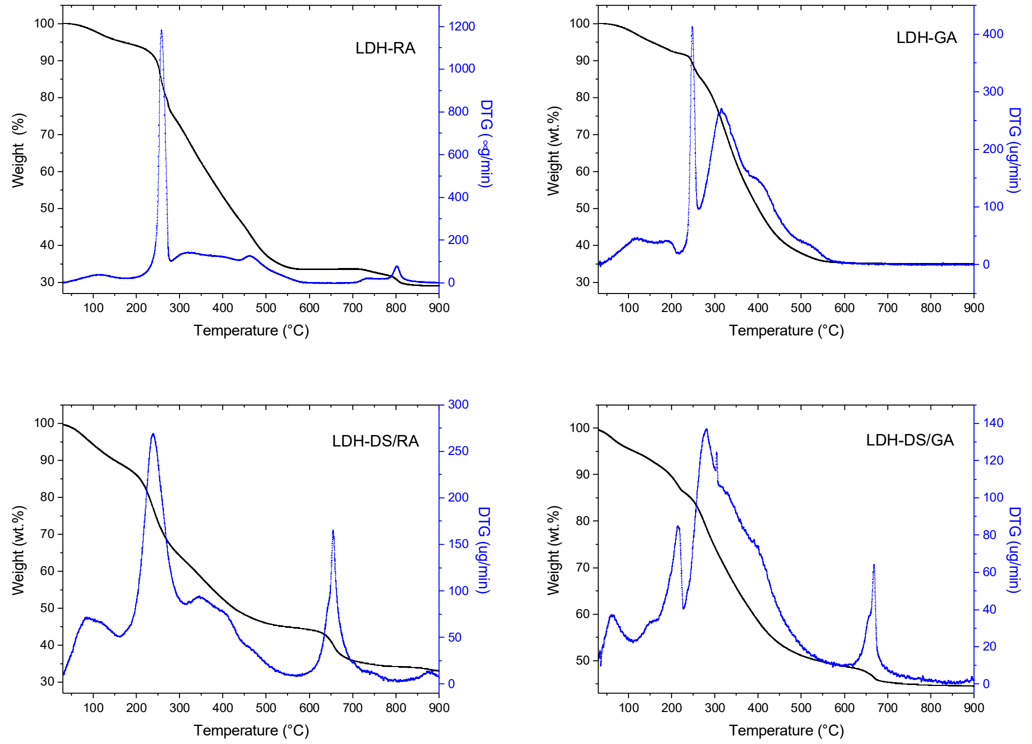

Figure S2. TGA and DTG curves of samples LDH-RA, LDH-GA, LDH-DS/RA, and LDH-DS/GA.

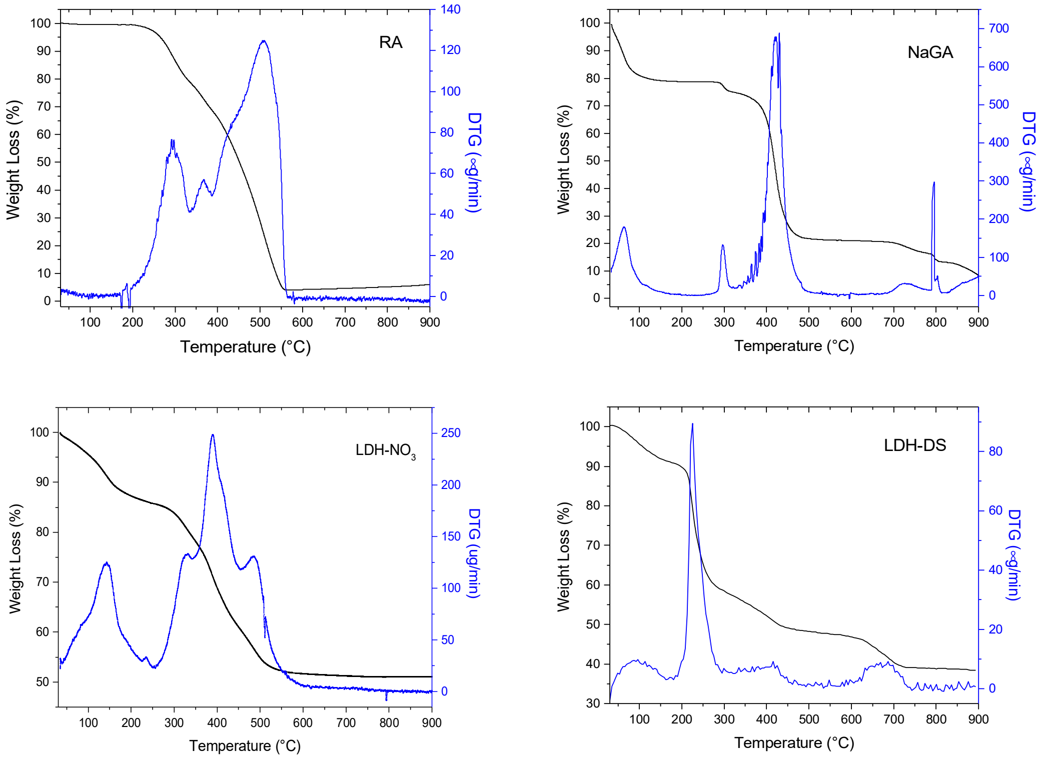

Figure S3. TGA and DTG curves of rosmarinic acid, NaGA, $LDH-NO_3$, and LDH-DS

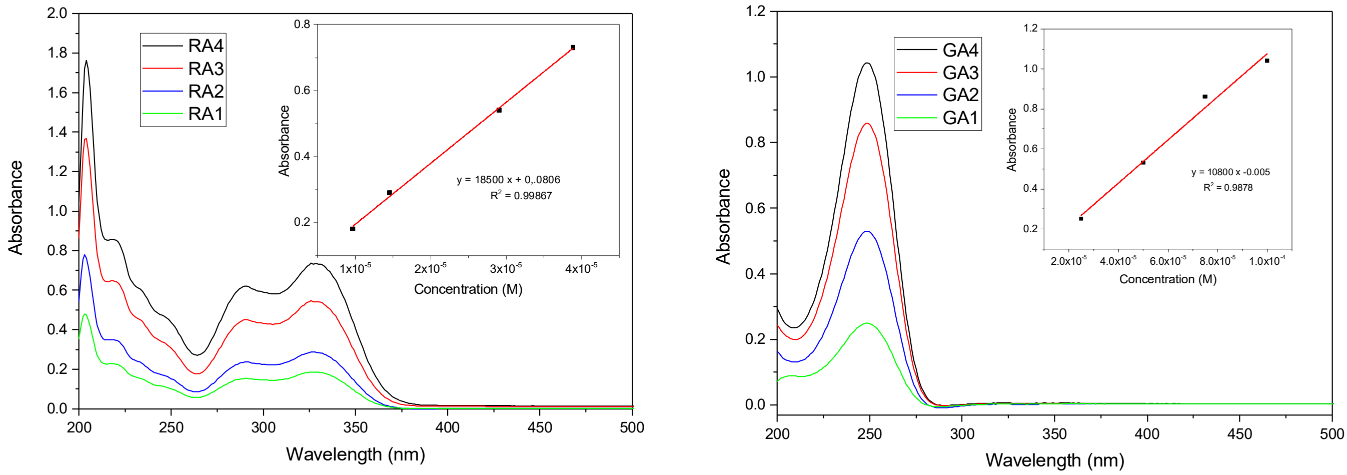

(**a**) (**b**)

Figure S4. UV-vis spectra of rosmarinic acid ethanol solutions at different concentrations and calibration curve (**a**) and UV-vis spectra of glycyrrhetinic acid ethanol solutions at different concentrations and calibration curve (**b**)

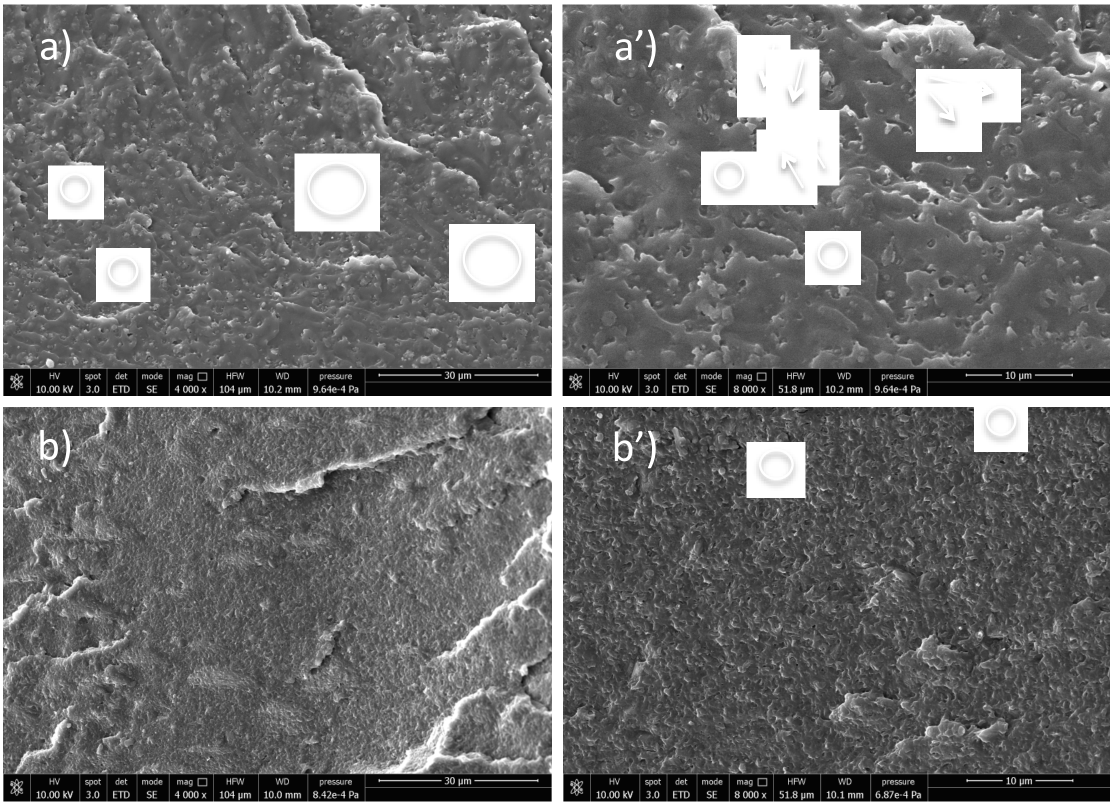

Figure S5. SEM images at different magnification of PLA/LDH-RA (a and a′) and PLA/LDH-GA (b and b′).

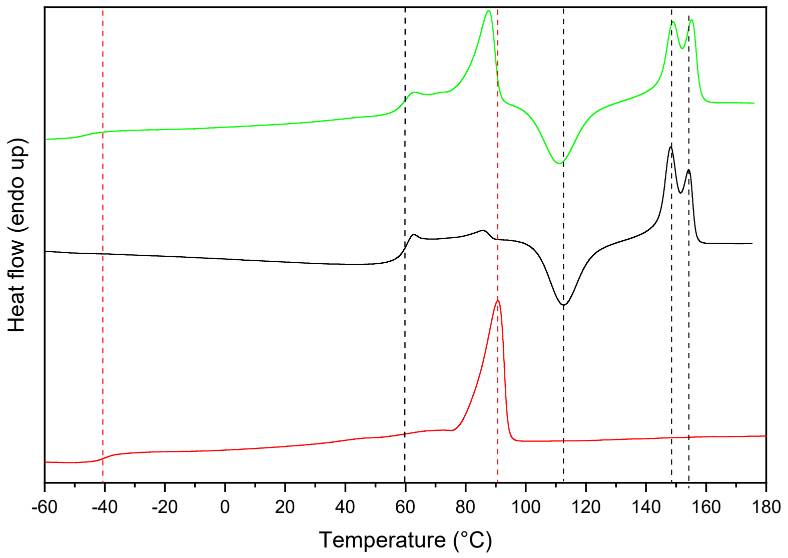

Figure S6. DSC curves of PLA (black), PBS (red) and PLA/PBS blend (green) (second heating). Red and black dotted lines indicate the fundamental transitions of pure PBS and PLA, respectively.

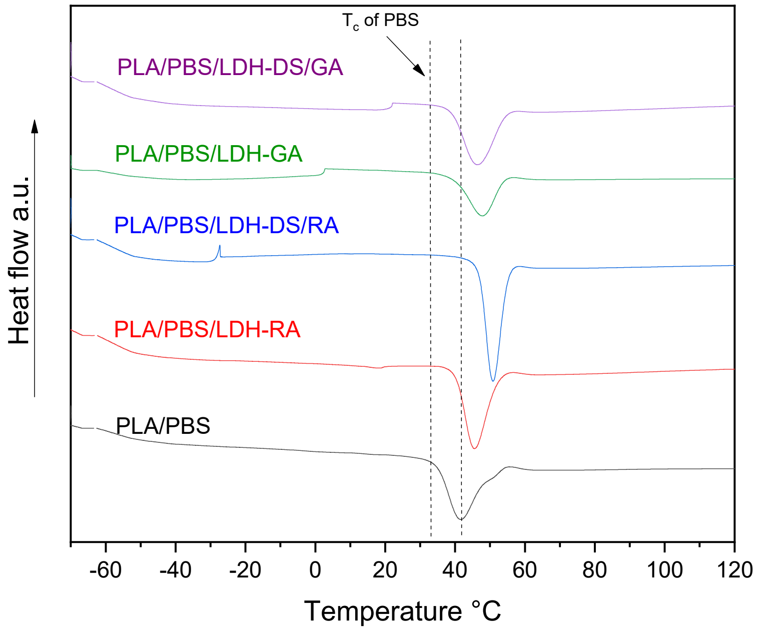

Figure S7. DSC curves (cooling) of PLA/PBS blend and its composites. Black dotted lines indicate the fundamental transitions of PLA/PBS blend.

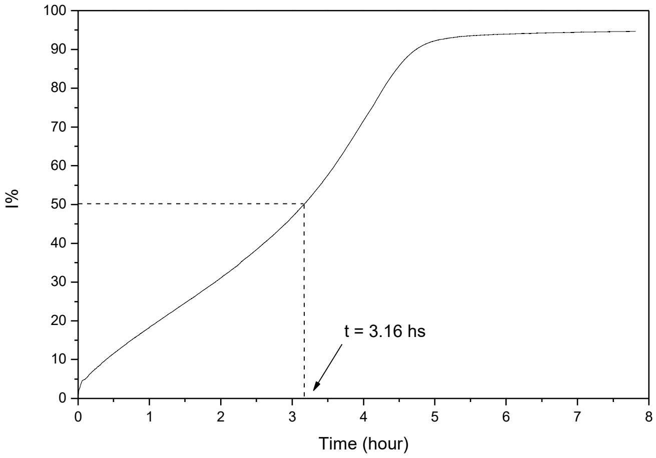

Figure S8. I% as a function of time for PLA/LDH-RA film in contact with DPPH stock solution.

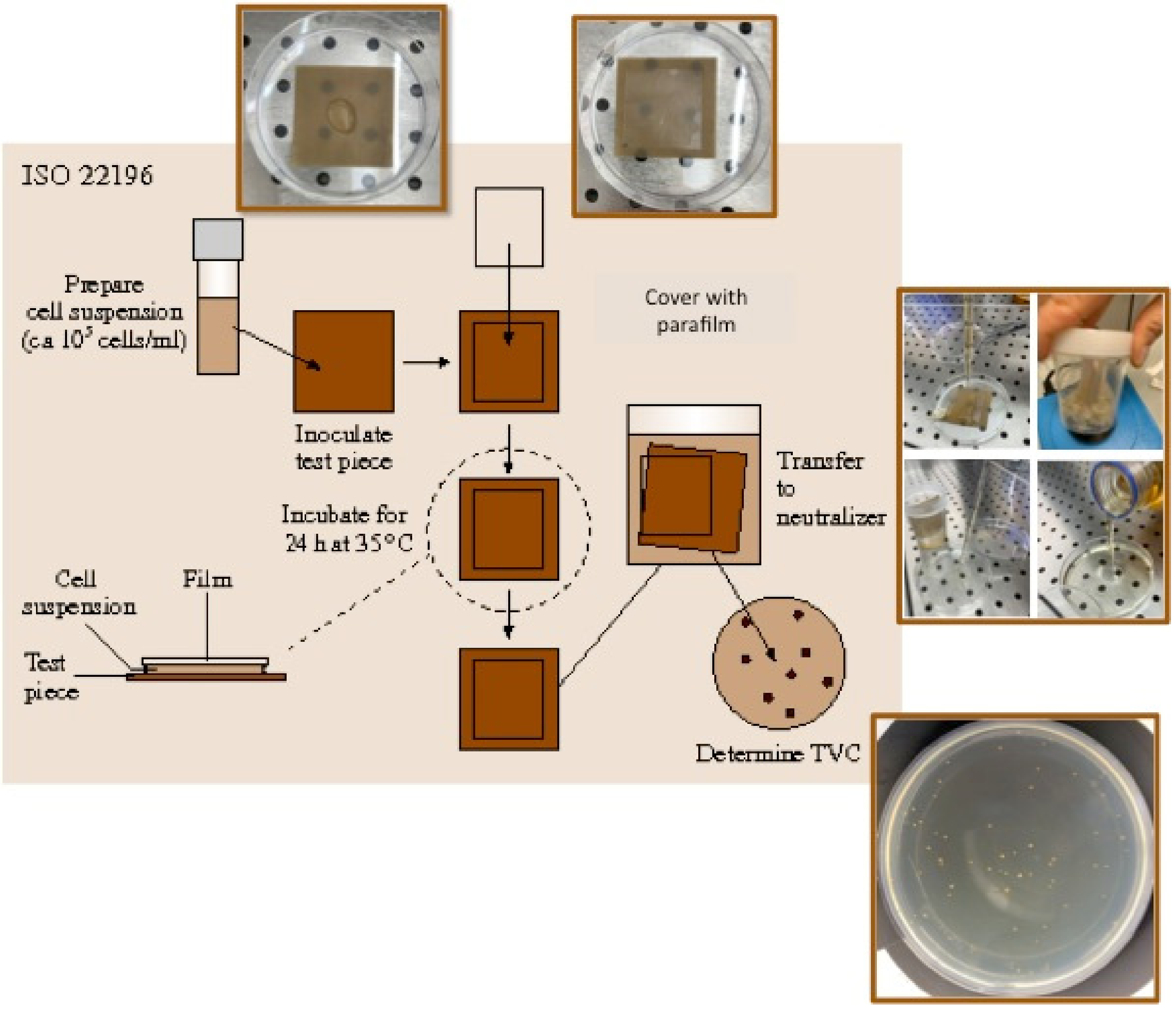

Figure S9. Schematic representation of antibacterial test by standard method ISO 22196:2011 with pictures registered during the experiment.

Table S1. Molecular weight and distribution of composites and their blanks

Sample	M_n (g mol^{-1})	M_w (g mol^{-1})	$Đ$[1]
PLA	157000	97600	1.6
PLA/LDH-RA	173000	106500	1.6
PLA/LDH-DS/RA	170100	114000	1.5
PLA/LDH-GA	168200	100100	1.7
PLA/LDH-DS/GA	155600	84200	1.8
PBS	140800	69600	2.0
PBS/LDH-RA	146300	70500	2.1
PBS/LDH-DS/RA	154500	74300	2.1
PBS/LDH-GA	127400	61900	2.1
PBS/LDH-DS/GA	133700	62300	2.1

[1] Dispersity (Đ) = Mw/Mn.

Table S2. DSC results for PLA, PBS, PLA/PBS and their composites.

Sample	T_g [a] (°C)		T_{cc} [b] (°C)		ΔH_{cc} [c] (J g^{-1})		T_{m1}, T_{m2} [d] (°C)		ΔH_m [e] (J g^{-1})		X_c [f] (%)	
	PLA	PBS	PLA	PBS	PLA	PBS	PLA	PBS	PLA	PBS	PLA	PBS
PLA	59	-	113	-	-24.8	-	148<br>154	-	26.7	-	28.7	-
PLA/LDH-RA	60	-	121	-	-32.9	-	151	-	23.7	-	26.8	-
PLA/LDH-DS/RA	59	-	129	-	-3.5	-	152	-	3.3	-	3.7	-
PLA/LDH-GA	60	-	126	-	-5.8	-	152	-	5.4	-	6.1	-
PLA/LDH-DS/GA	60	-	ND	-	ND	-	152	-	0.7	-	0.8	-
PBS	-	- 45	-	-	-	-	-	91	-	30.9	-	14.7
PBS/LDH-RA	-	- 43	-	-	-	-	-	86	-	38.5	-	18.4
PBS/LDH-DS/RA	-	- 45	-	-	-	-	-	86	-	31.8	-	15.2
PBS/LDH-GA	-	- 44	-	-	-	-	-	87	-	32.2	-	15.4
PBS/LDH-DS/GA	-	ND[g]	-	-	-	-	-	86	-	37.0	-	17.7
PLA/PBS	59	- 45	111	-	-14.2		149<br>155	87	13.0	10.8	23.3	12.9
PLA/PBS/LDH-RA	59	- 45	112	-	-15.2		149<br>155	87	13.7	11.3	25.7	13.5
PLA/PBS/LDH-DS/RA	59	- 45	112	-	-17.2		149<br>155	86	15.8	12.8	29.7	15.3
PLA/PBS/LDH-GA	58	- 45	124	-	-13.1		151	86	8.5	6.8	15.9	8.1
PLA/PBS/LDH-DS/GA	58	- 45	120	-	-17.1		151	86	14.8	8.2	27.8	9.8

[a] Glass transition temperature taken at middle point. [b] Cold crystallization temperature. [c] Cold crystallization enthalpy. [d] Melting temperature. [e] Melting enthalpy. [f] Degree of crystallization calculated with the following equation $X_c = \frac{1}{X_{polymer}} \times \frac{\Delta H_m}{\Delta H_m^0} \times 100$, where: $X_{polymer}$ = polymer weight fraction; ΔH_m = enthalpy associated to the melting process (when a multimodal melting peak is present, its total area is used); ΔH_m^0 is the enthalpy associated to the melting process of 100% crystalline PLA (93.1 J/g) or PBS (220 J/g); [g] No Detect

Migration test

Migration test of mono-deprotonated rosmarinic and glycyrrhetinic anions from films of PLA/LDH-RA and PLA/LDH-GA, respectively, was carried out by putting in contact 25 mg of film for each sample with 3 mL of EtOH/H_20 95/5 (v/v) at room temperature and stirring at 300 rpm. The release kinetics of rosmarinate and glycyrrhetinate anions was followed by UV-vis spectroscopy by recording UV-vis spectra of solutions at different time. For comparison, two samples of PLA mixed with RA or GA in the same amount present in the composites were prepared by solution mixing and used for migration tests.

Antioxidant activity

The radical scavenging activity of Trolox, RA, LDH-RA and LDH-DS/RA was determined according to the DPPH method. Stock methanol solutions of DPPH ($6x10^{-5}$ M), RA ($1x10^{-3}$ M), Trolox ($1.8x10^{-3}$ M) and a suspension of LDH-RA or LDH-DS/RA (1 mg in 2 mL) were prepared. In the case of LDH-RA and LDH-DS/RA the suspension was sonicated using a Hielscher Ultrasonic Processor UP200St for 10 min in order to favor the delamination of the organophilic LDH. In the case of RA: to 3 ml of DPPH solution, different aliquots (from 100 µL to 5 µL) of the RA stock solution and methanol were added to have the final volume of 3.1 mL, a blank DPPH solution was prepared by adding to 3 mL of DPPH stock solution, 100 µL of MeOH. The final concentration of RA in the DPPH solution was from about $3x10^{-5}$ M to $1.6x10^{-6}$ M. Analogously, in the case of Trolox: to 3 ml of DPPH solution different aliquots (from 50 µL to 5 µL) of stock Trolox solution and MeOH were added in order to have final volume of 3.05 mL and concentration of Trolox between $3x10^{-5}$ M to $5x10^{-6}$ M. Analogously, in the case of LDH-RA or LDH-DS/RA, the concentration of RA used in the DPPH solution corresponds to that of the rosmarinate anions present in the hybrid as determined from UV measurements: to 3 mL of DPPH solution different aliquots (from 90 µL to 5 µL) of LDH-RA or LDH-DS/R suspension and MeOH were added in order to have the final volume of 3.09 mL and final concentration of RA (considering that 37 wt.% of LDH-RA is RA and 13% of LDH-DS/RA is RA) between $1.5x10^{-5}$ M and $8x10^{-7}$ M. All solutions were maintained in the dark for 24 h then UV-vis spectra were recorded. For each sample the analysis was repeated two times, and average values of parameters as well as standard deviation were reported. The reduction percentage of the DPPH solution (I%) (Eq. 1) as a function of antioxidant concentration was reported and linear fitting of the experimental data was carried out. From this fitting, the EC_{50} value was calculated as the concentration of antioxidant that correspond to I% = 50%.

$$I\ \% = \frac{(A_0 - A_t)}{A_0} \times 100 \qquad \text{Eq. (1)}$$

where:

A_0 is the absorbance of the DPPH solution in the absence of antioxidant

A_t is the absorbance of the DPPH solution in the presence of antioxidant at the end of the reaction.

DPPH test was also used to evaluate the antioxidant power of the migrated fraction from PLA/LDH-RA film and the antioxidant ability of PLA/LDH-RA film. In the first case, the ethanolic solution resulting from migration of PLA/LDH-RA was dried to vacuum and to the residue, 3 mL of stock solution of DPPH was added and after 24 h in the dark UV-vis spectrum of the solution was recorded. In this case the I% was reported. Finally, to test the antioxidant power of PLA/LDH-RA film, 25 mg of the film were put in contact with 3 mL of stock DPPH solution and absorbance at 515 nm was recorded as a function of time.

Antibacterial activity

These tests were carried out onto PLA samples containing 4 wt% of LDH-RA or LDH-GA and were prepared in the melt by using an internal batch mixer (Brabender Plastograph OHG47055) with a chamber of 30 mL. Torque and temperature data were acquired by Brabender Mixing software Win-Mix ver.1.0. For each type of LDH, two LDH/PLA master-batches were prepared. First, 1 g LDH-RA or LDH-GA was suspended in chloroform (25 ml), stirred and probe-sonicated for 30 min. Then 1 g of PLA was dissolved in 15 ml of chloroform. The LDH suspension was added dropwise to the PLA solution and then the sample was recovered by solvent evaporation under vacuum and finally dried under vacuum at 50°C for 24 hours. Later, PLA/LDH composites were obtained by diluting the master-batches with PLA. In a typical experiment 23 g of PLA and 2 g of master-batch were added in the Brabender chamber at 180°C and mixed at 50 rpm for 10 min. Samples prepared in Brabender were coded as: B_PLA, B_PLA/LDH-RA, B_PLA/LDH-GA. Films of 100 µm thickness were obtained for each sample by compression molding using a Carver press model 4386 (Wabash, IN, USA) preheated at the temperature of 180 °C and used for further analysis. Antibacterial activity tests were performed

on two bacterial species, Escherichia coli and Staphylococcus aureus, following the standard method ISO 22196:2011. As control specimen, untreated PLA was used. Testing was performed on 3 replicates from each treated test material (i.e., films of B_PLA/LDH-RA and B_PLA/LDH-GA) and 6 specimens of the untreated material as control samples. 3 untreated test specimens were used to measure viable cells immediately after inoculation and 3 were used to measure viable cells after incubation for 24 h. Flat 50 x 50 mm specimens of the treated and untreated test materials were prepared with using sterile tools to avoid contamination with microorganisms or extraneous organic debris. As bacterial inoculum, 24 hours-old cultures of Escherichia coli and Staphylococcus aureus were used to prepare two solutions (1/500 Nutrient Broth) containing 5 x 105 cells/mL of each bacterial strain. Bacterial suspensions were immediately used for test assessment. Each test specimen was placed in a separate sterile 90 mm Petri dish with the test surface uppermost, then 0.4 mL of the test inoculum were placed on the test surface and covered with a 40 x 40 mm piece of parafilm that was gently pressed down to spread test inoculum to the edges, avoiding that the test inoculum leaked beyond the edges of the film. After the specimen was inoculated and covered with parafilm, the lid of the Petri dish was replaced and incubated at a temperature of 35 ± 1°C and a relative humidity of not less than 90 % for 24 ± 1 h. Immediately after inoculation, half of the untreated test specimens were processed by adding 10 ml of either SCDLP broth to the Petri dish containing the test specimen. Values obtained were used to determine the recovery rate of the bacteria from the test specimens. To ensure that the neutralizer completely washed the specimens, the SCDLP broth was collected and released at least four times using a pipette. After this treatment, specimens and SCDLP broth were transferred into a 100 ml sterile plastic pot and mechanically agitated by vortexing for 1 minute. SCDLP broth from pots was immediately used for viable bacteria enumeration. After 24-hour incubation, the remaining test specimens were processed as described above. Viable bacteria were enumerated by performing 10-fold serial dilutions of the SCDLP in phosphate-buffered physiological saline. 1 mL of each sample and their dilutions was placed into separate sterile Petri dishes and added with 15 mL of plate count agar. All plating was performed in duplicate. Petri dishes were incubated at 35 ± 1°C for 48 h. After incubation, the number of colonies in the Petri dishes was counted and recorded.

For the calculation of antibacterial activity, data are expressed as colony forming units (CFU) o viable cells for cm^2. The antibacterial activity was calculated using equation (2):

$$R = (U_t - U_0) - (A_t - U_0) = U_t - A_t \qquad \text{Eq. (2)}$$

where R is the antibacterial activity; U_0 is the average of the common logarithm (i.e. base 10 logarithm) of the number of viable bacteria, in cells/cm^2, recovered from the untreated test specimens immediately after inoculation; Ut is the average of the common logarithm of the number of viable bacteria, in cells/cm^2, recovered from the untreated test specimens after 24 h; At is the average of the common logarithm of the number of viable bacteria, in cells/cm^2, recovered from the treated test specimens after 24 h.
